# On the Importance of Ligand-Centered Excited States in the Emission of Cyclometalated Ir(III) Complexes

**DOI:** 10.1021/acs.inorgchem.1c01604

**Published:** 2021-08-16

**Authors:** Iván Soriano-Díaz, Enrique Ortí, Angelo Giussani

**Affiliations:** Instituto de Ciencia Molecular, Universidad de Valencia, Catedrático José Beltrán 2, 46980 Paterna, Spain

## Abstract

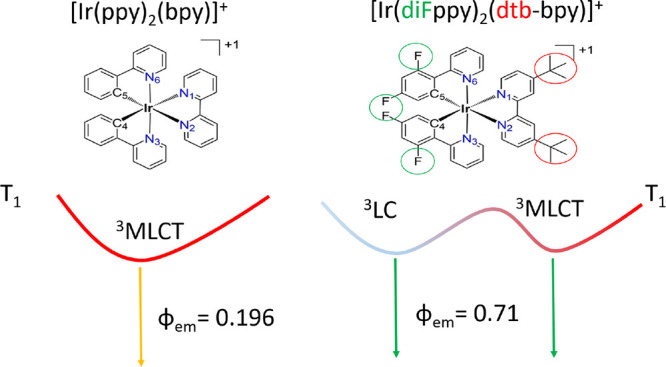

The photophysical behavior of the cyclometalating Ir(III) complexes [Ir(ppy)_2_(bpy)]^+^, where Hppy is 2-phenylpyridine and bpy is 2,2′-bipyridine (complex **1**), and [Ir(diFppy)_2_(dtb-bpy)]^+^, where diFppy is 2-(2,4-difluorophenyl)pyridine and dtb-bpy is 4,4′-di-*tert*-butyl-2,2′-bipyridine (complex **2**), has been theoretically investigated by performing density functional theory calculations. The two complexes share the same molecular skeleton, complex **2** being derived from complex **1** through the addition of fluoro and *tert*-butyl substituents, but present notable differences in their photophysical properties. The remarkable difference in their emission quantum yields (0.196 for complex **1** in dichloromethane and 0.71 for complex **2** in acetonitrile) has been evaluated by characterizing both radiative and nonradiative decay paths. It has emerged that the probability of decaying through the nonradiative triplet metal-centered state, normally associated with the loss of the emission quantum yield, does not appear to be the reason behind the reported substantially different emission efficiency. A more critical factor appears to be the ability of complex **2** to emit from both the usual metal-to-ligand charge-transfer state and from two additional ligand-centered states, as supported by the fact that the respective minima belong to the potential energy surface of the lowest triplet T_1_ state and that their phosphorescence lifetimes are in the same order of magnitude. In contrast, the emission of complex **1** can be originated only from the metal-to-ligand charge-transfer state, being the only emissive T_1_ minimum. The results constitute a significant case in which the emission from ligand-centered states is the key for determining the high emission quantum yield of a complex.

## Abstract 2

The reasons behind the significant increase in the emission quantum yield of the [Ir(diFppy)_2_(dtb-bpy)]^+^ complex with respect to [Ir(ppy)_2_(bpy)]^+^ are rationalized on the basis of DFT, TDDFT, and TDDFT-SOC calculations, revealing the key role that low-lying LC states can play in the emission properties of the complex.

## Introduction

Light-emitting electrochemical cells (LECs) based on ionic transition-metal complexes (iTMCs) represent a promising alternative in the development of highly efficient electroluminescent devices.^1,2^ LECs are particularly attractive in lighting applications because, in contrast to organic light-emitting diodes (OLEDs), they have the advantage of a much simpler structure that does not require rigorous encapsulation, which in turn drastically reduces the manufacturing costs.^3^ However, research on iTMCs for LEC applications is still facing different problems, mostly related to the need of obtaining complexes capable of emitting in a wide range of colors and having a high phosphorescence quantum yield.^4−11^ The last property, of crucial importance for the efficiency of a device, is the result of the competition between phosphorescence emission and all other possible relaxation mechanisms operating in a complex.

It is well-known in chemistry that the properties of a molecule can be significantly changed by introducing electronically active substituents.^12^ iTMCs for electroluminescent applications are not an exception, and a large part of the research conducted in the field has been attempted to fine-tune the photophysical properties of reference electroluminescent complexes. Among the heteroleptic cyclometalated Ir(III) complexes studied for electroluminescent applications, the [Ir(ppy)_2_(bpy)]^+^ complex, where Hppy is 2-phenylpyridine and bpy is 2,2′-bipyridine (hereafter complex **1**), is one of such reference systems.^13,14^ Many efforts have been devoted, both experimentally and theoretically, to elucidate its photophysical properties.^6,9,15−17^ Its emission quantum yield is far from the ideal value of one, being, for example, equal to 0.196 in dichloromethane at 298 K.^6^ Different modifications of the complex by changing the structure of the ligands and/or introducing electronically active substituents have been investigated in order to increase the emission quantum yield. One of the most successful modifications was achieved through the introduction of fluoro and *tert*-butyl groups, giving rise to the [Ir(diFppy)_2_(dtb-bpy)]^+^ complex, where diFppy is 2-(2,4-difluorophenyl)pyridine and dtb-bpy is 4,4′-di-*tert*-butyl-2,2′-bipyridine (hereafter complex **2**). For [Ir(diFppy)_2_(dtb-bpy)]^+^, a noticeable value of 0.71 was measured for the emission quantum yield in acetonitrile at 298 K.^9,18^

A common mechanism through which iTMCs have been shown to decay in a nonradiative way is caused by the possible population of metal-center (MC) triplet states.^19−21^ The ^3^MC states lead the system to a strong geometrical deformation, normally associated with a significant stretching of the metal–ligand bonds, and are characterized by equilibrium structures in which the energy gap with the ground state is drastically reduced and from which crossing regions with the ground state are in general easily accessible. Heteroleptic cyclometalated Ir(III) complexes as [Ir(ppy)_2_(bpy)]^+^-type complexes are also suffering from such a nonradiative mechanism,^22,23^ as, for example, discussed in the work of Accorsi, Ortí and co-workers.^6^ In that work, the decrease in the emission quantum yield of [Ir(ppy)_2_(bpy)]^+^ derivatives obtained though the addition of phenyl rings on the bpy ligand was rationalized by the increase in the decay probability through an MC-mediated nonradiative path.^6^

In the present contribution, the photophysical properties of the [Ir(ppy)_2_(bpy)]^+^ and [Ir(diFppy)_2_(dtb-bpy)]^+^ complexes have been studied by performing density functional theory (DFT), time-dependent DFT (TDDFT), and TDDFT spin–orbit coupling (TDDFT-SOC) calculations. The goal was to understand the molecular reasons behind the reported significant difference in their emission quantum yields (0.196 and 0.71, respectively),^6,9^ in order to help in establishing molecular design criteria aimed at augmenting the emission efficiency of cyclometalated Ir(III) complexes. On the basis of the theoretical characterization performed, it has emerged that a different involvement of nonradiative MC states in the photophysics of both systems is not a plausible cause leading to their different emission efficiency. Instead, the presence of three different emission minima in complex **2**, one associated to the usual metal-to-ligand charge-transfer (MLCT) state and two related to ligand-centered (LC) states, whereas only one emissive MLCT state is obtained in complex **1**, appears as the key factor determining the much higher emission quantum yield of complex **2** with respect to complex **1**.

## Methods and Computational Details

DFT calculations were performed employing Becke’s three-parameter B3LYP exchange-correlation functional^24,25^ and using the 6-31G** basis set for C, H, N, and F^26^ and the “double-ζ” quality LANL2DZ basis set for the Ir atom.^27^ The inner core electrons of Ir were substituted by an effective core potential while explicitly treating the outer core [(5s)^2^(5p)^6^] and the (5d)^6^ valence electrons. Geometry optimizations of minima and transitions states were performed with Gaussian without imposing any symmetry restriction. Gaussian optimization of transition states was performed using the Berny algorithm, and the corresponding IRC paths were obtained in order to verify the connection with the correct minima. The spin-unrestricted UB3LYP approach with a spin multiplicity of 3 was used to compute triplet states. Emission energies were calculated in all triplet minima belonging to the lowest-energy triplet (T_1_) potential energy surface (PES), and they were estimated as the energy difference between the triplet state and the ground state at the corresponding T_1_ minimum. TDDFT calculations of the triplet excited states were performed at the B3LYP/(6-31G**+LANL2DZ)-optimized equilibrium geometry of the electronic ground state (S_0_). TDDFT calculations were also performed in order to optimize triplet states higher than T_1_. All the above described calculations were performed using the Gaussian 16 program.^28^

Additional DFT and TDDFT-SOC calculations were conducted using the Orca 4.1.2 software.^29,30^ These calculations were performed employing a ZORA Hamiltonian in order to account for relativistic effects,^31^ a ZORA-DEF2-TZVP basis set for C, H, N, and F, and a SARC-ZORA-TZVP basis set for Ir.^32^ A mean-field spin–orbit operator was used in the ORCA calculations. The Orca software was employed for the optimization of minimum-energy crossing points (MECPs)^33,34^ for singlet/triplet crossing (STC) and for performing TDDFT-SOC calculations accounting for the spin–orbit coupling (SOC) between singlet and triplet states. The latter calculations were performed in all T_1_ minima by computing 25 singlet and 25 triplet states. The resulting energies and dipole moments including SOC interactions were used for obtaining the radiative rate *k*_rad_ employing the following equation^35,36^
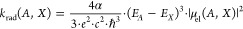
1where *A* indicates the state from which the radiation is produced, *X* is the ground state, α is the fine-structure constant, *e* is the electron charge, *c* is the speed of light, *ℏ* is the reduced Planck constant, and μ_el_ is the electric dipole transition moment. The so-obtained radiative rates were corrected for the refractive index *n* of the solvent by multiplying their values by the square of *n* (*n* = 1.42 and 1.34 for dichloromethane and acetonitrile, respectively). For each T_1_ minimum, the phosphorescence lifetime τ(T_1_) was finally obtained using the following equation
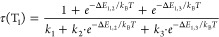
2where *k*_1_, *k*_2_, and *k*_3_ are the radiative rates of the three sublevels in which the T_1_ state splits, Δ*E*_1,2_ is the energy difference between sublevels 2 and 1, Δ*E*_1,3_ is the energy difference between sublevels 3 and 1, *k*_B_ is the Boltzmann constant, and *T* is the temperature.

For all the calculations performed with Gaussian, solvent effects (dichloromethane for complex **1** and acetonitrile for complex **2**) were taken into account by employing the polarized continuum model (PCM) method.^37^ The computations performed with Orca, MECP optimizations and TDDFT-SOC calculations, were instead performed in the gas phase, although the final energies of the optimized MECP points were then recomputed with Gaussian using the PCM model. To validate the use of two different levels of theory with two different codes, B3LYP/(6-31G**+LANL2DZ) with Gaussian and B3LYP/(ZORA-DEF2-TZVP+SARC-ZORA-TZVP) with Orca, a comparison of the two approaches was made. In particular, the energies of the excited states calculated for the geometries in which the Orca code was employed for computing the radiative lifetimes were compared (see the Results and Discussion section and Table S1).

## Results and Discussion

### Radiative and Nonradiative Decays for Complex **1**

**Figure 1:**
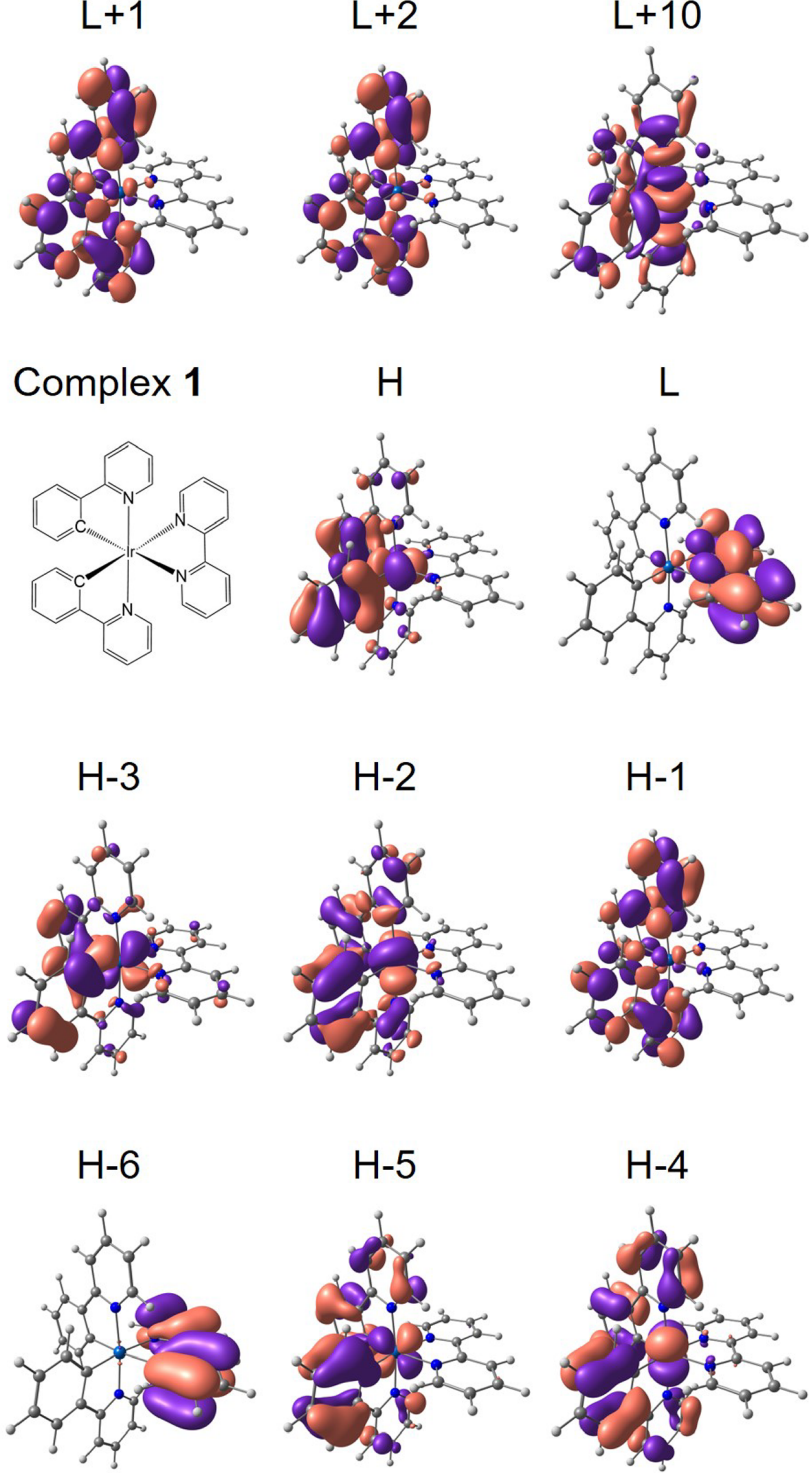
Isovalue contour plots (±0.03 a.u.) computed for the molecular orbitals of complex **1** at the DFT B3LYP/(6-31G**+LANL2DZ)-PCM level. H and L denote HOMO and LUMO, respectively. The analogous data for complex **2** are reported in Figure S7.

**Figure 2:**
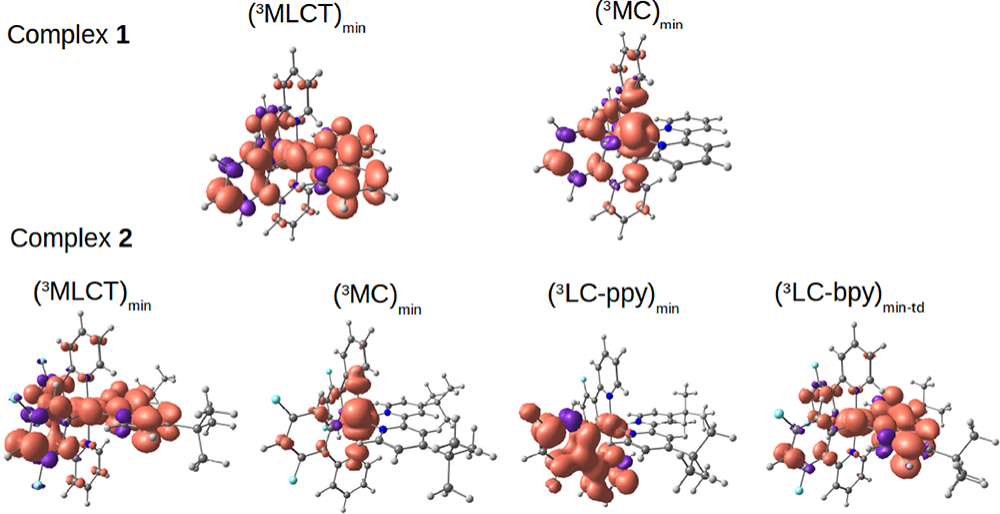
Unpaired-electron spin density contours (0.002 a.u.) calculated at the DFT-B3LYP/(6-31G**+LANL2DZ)-PCM level for the different triplet states of complexes **1** and **2** at their respective optimized minima.

**Figure 3:**
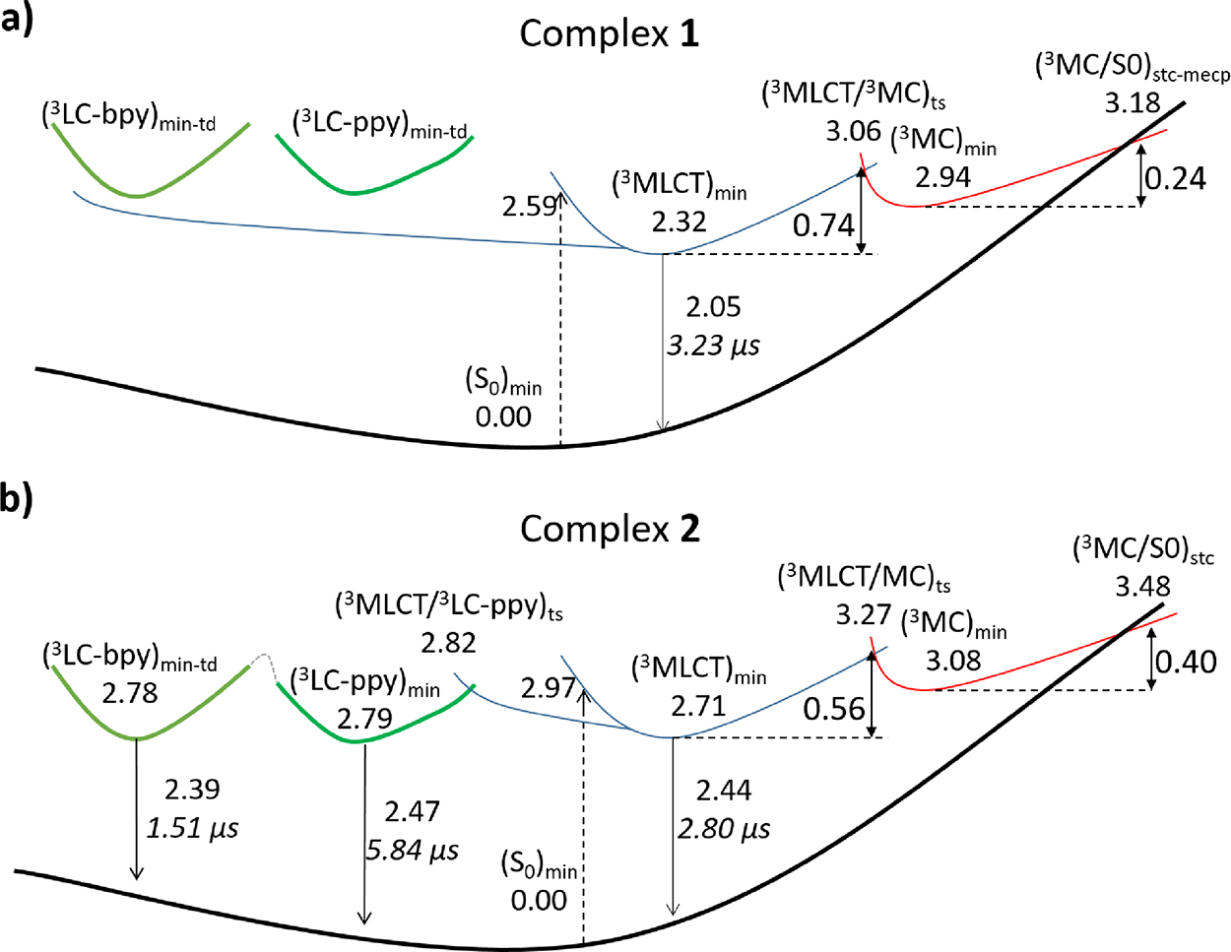
Schematic representation of the characterized photophysics of complexes **1** (a) and **2** (b). All the reported energies are in eV and have been computed at the DFT B3LYP/(6-31G**+LANL2DZ)-PCM level (dichloromethane for **1** and acetonitrile for **2**). The phosphorescence lifetimes calculated for the T_1_ minima are also reported in μs (italics).

The ground-state geometry of complex **1** was optimized at the DFT B3LYP/(6-31G**+LANL2DZ)-PCM level using CH_2_Cl_2_ as a solvent. The resulting S_0_ minimum, hereafter (S_0_)_min_, displays a near-octahedral coordination for the Ir metal, and the obtained geometrical parameters are in agreement with the theoretical and experimental X-ray data available in the literature (see ref (6), Table 1, and Figures S1–S3 in the Supporting Information). At the (S_0_)_min_, the lowest triplet state computed at the spin-unrestricted UDFT level is placed 2.59 eV above the S_0_ state. Through the analysis of the DFT orbitals and by the examination of the dominant monoexcitations obtained out of a TDDFT computation at (S_0_)_min_, it is possible to conclude that such a triplet state has an MLCT electronic nature, mixed with some ligand-to-ligand charge-transfer (LLCT) character. The state is in fact described by one main electronic monoexcitation from the HOMO to the LUMO (Table 2), the former being localized on the phenyl rings of both ppy ligands and on the Ir atom, whereas the latter is mostly localized on the bpy ligand (Figure 1). The DFT spin density associated to the two unpaired electrons confirms the MLCT/LLCT nature of the state (see Figure 2). Such a state will hereafter be denoted as ^3^MLCT. Starting from the (S_0_)_min_ geometry, the ^3^MLCT state was additionally optimized at the UDFT level. The corresponding ^3^MLCT minimum, hereafter (^3^MLCT)_min_, preserves the near-octahedral conformation, and the geometry appears to be very similar to that of the (S_0_)_min_ (Table 1). At the DFT level, the ^3^MLCT state in its minimum is 2.32 eV above the S_0_ state at the (S_0_)_min_ geometry, whereas its emission energy, calculated as the vertical energy with respect to the ground state at the (^3^MLCT)_min_ geometry, is equal to 2.05 eV. The latter value, corresponding to a wavelength of 605 nm, is in agreement with the emission maximum recorded in dichloromethane at 298 K (595 nm, 2.08 eV),^6^ thus supporting the ^3^MLCT state as responsible for the experimentally recorded emission of complex **1**. The so-described radiative decay path (*i.e.*, population of the ^3^MLCT state, decay to its corresponding minimum, and radiative emission) is represented in the central part of Figure 3a.

**Table 1:**
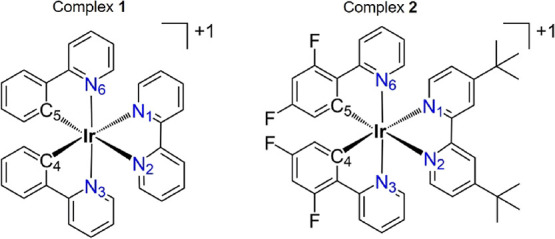
DFT B3LYP/(6-31G**+LANL2DZ)-PCM Optimized Bond Lengths (in Å) Computed for the Critical Points Characterized for Complexes **1** and **2**

Complex **1**						
bond/geometry	(S_0_)_min_	(^3^MLCT)_min_	(^3^MC)_min_	(^3^MC/S_0_)_stc-mecp_	(^3^LC-ppy)_min-td_^a^	(^3^LC-bpy)_min-td_^a^
Ir–N1	2.209	2.195	2.224	2.206	2.256	2.172
Ir–N2	2.209	2.195	2.224	2.203	2.256	2.172
Ir–N3	2.083	2.081	2.507	2.819	2.076	2.085
Ir–C4	2.023	1.999	2.016	1.998	1.992	2.030
Ir–C5	2.023	1.999	2.016	1.997	1.992	2.030
Ir–N6	2.083	2.081	2.507	2.713	2.076	2.085

Complex **2**						
bond/geometry	(S_0_)_min_	(^3^MLCT)_min_	(^3^MC)_min_	(^3^MC/S_0_)_stc_	(^3^LC-ppy)_min_	(^3^LC-bpy)_min-td_^a^
Ir–N1	2.197	2.181	2.201	2.184	2.207	2.172
Ir–N2	2.197	2.177	2.201	2.183	2.216	2.158
Ir–N3	2.080	2.078	2.478	2.717	2.052	2.076
Ir–C4	2.021	2.002	2.040	2.012	1.998	2.022
Ir–C5	2.021	2.003	2.040	2.012	2.018	2.024
Ir–N6	2.080	2.078	2.478	2.900	2.090	2.078

a Geometry obtained at the TD-DFT B3LYP/(6-31G**+LANL2DZ)-PCM level.

**Table 2:** Low-Lying Triplet Excited States Calculated at the TDDFT B3LYP/(6-31G**+LANL2DZ)-PCM Level for Complexes **1** and **2**^a^

complex	state	*E*	monoexcitations	description
		(eV)	(%)	
**1**	T_1_ (^3^MLCT)	2.43	H → L (98)	MLCT/LLCT
	T_2_ (^3^LC-ppy)	2.75	H → L+1 (67)	LC
			H–1 → L+2 (16)	LC
	T_3_	2.80	H → L+2 (55)	LC
			H–1 → L+1 (25)	LC
	T_4_ (^3^LC-bpy)	2.93	H–2 → L (37)	MLCT
			H–6 → L (29)	LC
			H–4 → L (21)	MLCT
	T_5_	3.05	H–3 → L (45)	MLCT/LLCT
			H–1 → L (36)	LLCT
			H–5 → L (12)	MLCT/LLCT
	T_6_	3.19	H → L+2 (37)	LC/MLCT
			H–2 → L+2 (16)	LC/MLCT
			H–1 → L+1 (15)	LC
	T_30_ (^3^MC)	4.26	H → L+10 (50)	MC
**2**	T_1_ (^3^MLCT)	2.85	H → L (91)	MLCT/LLCT
	T_2_ (^3^LC-ppy)	2.89	H → L+1 (51)	LC/MLCT
			H–1 → L+2 (23)	LC/MLCT
			H–2 → L+1 (11)	LC/MLCT
	T_3_	2.92	H → L+2 (39)	LC/MLCT
			H–1 → L+1 (33)	LC/MLCT
			H–2 → L+2 (11)	LC/MLCT
	T_4_ (^3^LC-bpy)	3.05	H–6 → L (45)	LC
			H–3 → L (24)	MLCT
	T_5_	3.28	H–5 → L (48)	LLCT
			H–1 → L+2 (36)	LC/MLCT
	T_6_	3.34	H → L+1 (41)	LC/MLCT
			H–2 → L+1 (24)	LC/MLCT
			H–1 → L+2 (13)	LC/MLCT
	T_73_ (^3^MC)	5.33	H → L+10 (58)	MC

a Vertical excitation energies (*E*), dominant monoexcitations with contributions (within parentheses) greater than 10%, and electronic description of the excited state are reported. H and L denote HOMO and LUMO, respectively.

The emission quantum yield of complex **1** in dichloromethane at 298 K is equal to 0.196.^6^ This value highlights the presence of nonradiative decay paths that bring the system back to the initial ground state without light emission. A plausible nonradiative decay for Ir complexes involves the population of a nonradiative ^3^MC state, normally associated with a strong lengthening of some of the bonds coordinating the central Ir atom to the ligands.^22^ Complex **1** is not an exception, and a nonradiative MC-mediated decay was previously described for the complex,^6^ being characterized by a large elongation of the Ir–N_ppy_ bonds. Starting from an initial geometry obtained from the ^3^MLCT minimum through the elongation of the Ir–N_ppy_ bonds, a DFT optimization of the lowest triplet state ended in the minimum of a ^3^MC state, where the Ir–N_ppy_ bonds have lengthened from 2.083 Å in (S_0_)_min_ to 2.507 Å (Table 1). The MC nature of this state is confirmed by the inspection of the DFT spin density (Figure 2). In the ^3^MC minimum, hereafter (^3^MC)_min_, the MC state is the lowest triplet state and is placed 2.94 eV above the S_0_ state in its minimum, whereas its vertical emission energy is equal to 1.31 eV. Such a decrease in the energy gap with the S_0_ state is mainly due to the energy increase experimented by S_0_ (1.63 eV) at the (^3^MC)_min_ geometry, most likely associated with the significant structural deformation there displayed with respect to (S_0_)_min_ (∼0.4 Å elongation of the Ir–N_ppy_ bonds). It should be mentioned that TDDFT calculations place the ^3^MC state very high in energy (4.26 eV) at the (S_0_)_min_ geometry (Table 2).

From the ^3^MC minimum, a DFT T_1_/S_0_ MECP optimization was performed as implemented in Orca (see Computational Details). The optimization of MECPs using the Orca code was previously employed in the study of transition-metal complexes.^19−21,23^ As expected, the geometry of the resulting MECP is characterized by a further enlargement of the Ir–N_ppy_ bonds, which lengthen in an asymmetric way to 2.71 and 2.82 Å, respectively (Table 1). This indicates that the Ir–N_ppy_ distances are the key coordinates for reaching the crossing region, and a similar crossing point was indeed characterized upon performing a relaxed scan from the MC minimum by systematically elongating such bonds. The T_1_/S_0_ MECP, hereafter (^3^MC/S_0_)_stc-mecp_, is placed 3.18 eV above (S_0_)_min_, which means that from (^3^MC)_min_, an energy barrier of 0.24 eV must be surmounted to reach the crossing region (Figure 3a). The global barrier to decay through the (^3^MC/S_0_)_stc-mecp_ point is, however, not only related to the latter value but also includes the energy required for reaching the (^3^MC)_min_ structure from the lower and presumably initially populated ^3^MLCT equilibrium geometry. The transition state (TS) connecting the two T_1_ minima (*i.e.*, the ^3^MLCT and ^3^MC minima), hereafter (^3^MLCT/^3^MC)_ts_ (see Figure S8), was obtained at the DFT level and is placed 3.06 eV above (S_0_)_min_. Consequently, an energy barrier of 0.74 eV has to be overcome to go from (^3^MLCT)_min_ to (^3^MC)_min_. The nonradiative decay passing through the (^3^MC/S_0_)_stc-mecp_ geometry can then be framed as a two-step process, which first implies the evolution from the ^3^MLCT minimum to the ^3^MC minimum limited by a barrier of 0.74 eV and second involves the decay back to the ground state through the (^3^MC/S0)_stc-mecp_ crossing region with a barrier of 0.24 eV, as represented in the right part of Figure 3a. The first step appears consequently as the limiting step since a 0.74 eV barrier (17.06 kcal mol^–1^) is not a negligible barrier, especially considering that most of the excitation energy should decay in a nonradiative fashion, the emission quantum yield being equal to 0.196. The limitations of the adopted model should, however, be taken into account, as the employed level of theory, DFT, and the intermolecular quenching processes, known to play an important role in the photophysics of transition-metal complexes as the ones here studied,^38^ are not here simulated.

In order to have a more complete vision of complex **1** in the excited state, other triplet excited states apart from the ones above described were characterized. The TDDFT computation of the lowest triplet states of the system at the (S_0_)_min_ reveals that above the lowest-energy ^3^MLCT state, ^3^LC states appear close in energy (Table 2). The T_2_ and T_3_ states at 2.75 and 2.80 eV, respectively, are both LC states mainly localized on the ppy ligands, whereas state T_4_ at 2.93 eV is a mix between an LC associated to the bpy ligand and an MLCT excitation similar to the one characterizing the T_1_ state. Both the T_2_ and T_4_ states, hereafter ^3^LC-ppy and ^3^LC-bpy, respectively, were optimized at the TDDFT level (Table 1) due to the impossibility of employing DFT since neither of them resulted to be the lowest triplet state in any of the here explored geometries. The obtained minima will hereafter be named (^3^LC-ppy)_min-td_ and (^3^LC-bpy)_min-td_, respectively, where the subscript “td” indicates that the minima were obtained at the TDDFT level. The ^3^LC-ppy and ^3^LC-bpy states are 0.07 and 0.19 eV, respectively, higher in energy than the ^3^MLCT state at their corresponding minima (see TDDFT energies in Table S2). The results indicate that neither the ^3^LC-ppy nor the ^3^LC-bpy state is most likely involved in the complex emission since in their minima, the excitation can easily decay to the still lower in energy ^3^MLCT state. The so-described additional exploration of the PESs of complex **1**, although leading back to the ^3^MLCT minimum, is represented in the left part of Figure 3a as it will be useful for comparison with complex **2**.

### Radiative and Nonradiative Decay for Complex **2**

DFT calculations similar to those discussed above for complex **1** were performed for **2**. As for complex **1**, the ground-state minimum-energy geometry of complex **2** presents a near-octahedral structure (see Table 1 and Figures S4–S6) and the lowest triplet state, vertically placed at 2.97 eV (UDFT energy), and has mainly an MLCT nature describing a charge-transfer electronic promotion from the Ir(ppy)_2_ environment to the bpy ligand (see Table 2, Figure S7, and Figure 2). The DFT optimization of the ^3^MLCT state results in a minimum-energy geometry that preserves the near-octahedral structure and is placed 2.71 eV above the (S_0_)_min_ while being vertically placed at 2.44 eV with respect to S_0_. The latter value is in agreement with the experimental emission recorded in acetonitrile at 298 K (524 nm, 2.37 eV), which supports the involvement of the ^3^MLCT state in the emission of the complex. The so-described radiative decay path (*i.e.*, population of the ^3^MLCT triplet state, decay to the (^3^MLCT)_min_, and radiative emission) is represented in the central part of Figure 3b.

To characterize the nonradiative ^3^MC state, the latter was optimized at the UDFT level starting from a geometry obtained by elongating the Ir–N_ppy_ bond lengths of the ^3^MLCT minimum. The minimum of the ^3^MC state displays strongly elongated Ir–N_ppy_ bond distances, both equal to 2.48 Å (Table 1). The ^3^MC minimum is placed 3.08 above the (S_0_)_min_, and at the (^3^MC)_min_ geometry, the ^3^MC/S_0_ gap is drastically reduced to 1.54 eV, again due to the destabilization suffered by the S_0_ state. From the (^3^MC)_min_, an MECP calculation was attempted, but its convergence was not achieved. Nevertheless, geometries in which the ^3^MC and S_0_ states are energetically degenerate were localized along the MECP optimization. The lower one of them, hereafter (^3^MC/S_0_)_stc_, is placed 3.48 eV above the (S_0_)_min_, which means that the system has to surmount an energy barrier of 0.40 eV to reach such a crossing region from the ^3^MC minimum. The geometry features an even more pronounced elongation of the Ir–N_ppy_ bond distances, now equal to 2.90 and 2.72 Å. Since the characterized crossing is not a converged MECP, the 0.40 eV barrier is actually an upper bound of the energy required to arrive at the true MECP. In the nonradiative decay mediated by the (^3^MC/S_0_)_stc_ structure, the barrier separating the ^3^MLCT and ^3^MC minima must also be accounted for, which has been done through the characterization of the corresponding TS, (^3^MLCT/^3^MC)_ts_ (see Figure S9). The latter is placed 3.27 eV above the (S_0_)_min_, thus indicating a 0.56 eV energy barrier from the (^3^MLCT)_min_ structure. Consequently, as for complex **1**, the MC-mediated nonradiative decay of complex **2** can be framed as a two-step process, characterized respectively by 0.56 and 0.40 eV energy barriers (0.96 eV in total). The so-described nonradiative decay path (*i.e.*, the evolution from the ^3^MLCT minimum to the ^3^MC minimum and the subsequent decay back to the ground state through the (^3^MC/S_0_)_stc_ crossing region) is represented in the right part of Figure 3b.

It is interesting to notice that the magnitude of the energy barriers to attain the ^3^MC/S_0_ crossing region in the two complexes is comparable, their sum being almost equal (0.98 and 0.96 eV for complexes **1** and **2**, respectively), thus indicating a similar accessibility of the nonradiative decay in both complexes. The passage from the (^3^MLCT)_min_ to the (^3^MC)_min_ requires 0.74 and 0.56 eV for complexes **1** and **2**, respectively, meaning that the population of the nonradiative ^3^MC state is even more favorable in the highly emissive complex **2** than in complex **1**. In the second step, the decay to the ^3^MC/S_0_ crossing is more favorable for complex **1** than for complex **2**, the corresponding barriers being equal to 0.24 and 0.40 eV, respectively. The difference between the two barriers is not too high (0.16 eV) also considering that the barrier for complex **2** is actually an upper bound of the energy required for reaching the true MECP point.

From these results, we can conclude that a significantly different involvement of the MC-mediated nonradiative decay does not appear to be the reason behind the significant different emission quantum yield registered for the two complexes. It is, however, true that a smaller ^3^MC/S_0_ energy gap characterizes the ^3^MC minimum of complex **1** (1.31 eV) compared to complex **2** (1.54 eV), thus suggesting that a more efficient nonradiative vibronic decay occurs in complex **1** than in complex **2**, but again, such a difference does not appear to justify the large difference in the emission efficiency of the two systems.

A possible cause leading to the reported high emission quantum yield of complex **2** could be a higher efficiency in the radiative decay from the ^3^MLCT minimum. To investigate this efficiency, the phosphorescence lifetime of the ^3^MLCT state in its minimum was computed for the two complexes. TDDFT computations accounting for the SOC between singlet and triplet states were performed using the Orca software, and the phosphorescence lifetime was obtained using the expression for spontaneous emission (see Computational Details). In the ^3^MLCT minimum of complex **1**, among the computed 100 singlet-triplet mixed excited states that result from a TDDFT-SOC computation over 25 singlet and 25 triplet states, the three lowest excited states are indeed mainly composed of the triplet state having an MLCT nature (see Figure S11). It is in fact possible to label the singlet-triplet mixed states as either triplet-like (*i.e.*, a state mainly composed by a pure triplet state with some secondary contributions of other triplet or singlet states) or singlet-like. Using the data for such three triplet-like states and eq 2, the phosphorescence lifetime of the ^3^MLCT state of complex **1** is estimated to be 3.23 μs. It is interesting to note that the fourth state in energy is placed only 0.033 eV above the lowest excited state and is mainly described as a pure singlet state associated with the HOMO-to-LUMO electronic promotion. The same analysis was done for complex **2**, and the three sublevels of the ^3^MLCT state were recognized as the three lowest among the computed singlet-triplet mixed states (see Figure S12). The phosphorescence lifetime at the (^3^MLCT)_min_ of complex **2**, evaluated using the three sublevels of the ^3^MLCT state from the TDDFT-SOC computation, was computed to be equal to 2.80 μs. Again, the next state is mainly a HOMO-to-LUMO singlet state, and it is placed at only 0.031 eV above the lowest excited state. The phosphorescence lifetimes estimated for the ^3^MLCT state of complexes **1** and **2** do not appear to be significantly different and consequently cannot explain the remarkable high difference in the emission quantum yield of the two complexes. For both complexes, the appearance of a low-lying singlet-like excited state associated to the HOMO-to-LUMO excitation is not particularly surprising, considering that the singlet equivalent of the ^3^MLCT state (hereafter ^1^MLCT) is at all computed geometries almost degenerate with the ^3^MLCT state (see Table S2).

As for complex **1**, the ^3^LC-ppy and ^3^LC-bpy states were also localized for complex **2** as the T_2_ and T_4_ states at the (S_0_)_min_ geometry. In this case, however, the ^3^LC-ppy and ^3^LC-bpy states resulted to be the lowest triplet state at their respective TDDFT-optimized minima, (^3^LC-ppy)_min-td_ and (^3^LC-bpy)_min-td_. For the ^3^LC-ppy state, it was possible to reoptimize the TDDFT geometry at the UDFT level, obtaining the structure hereafter denoted as (^3^LC-ppy)_min_. The minimum preserves the near-octahedral structure, and the main changes with respect to the (S_0_)_min_ geometry are indeed localized on the ppy ligand, in agreement with the nature of the state (see Figures S4–S6). The nature of the state was further confirmed through the computation of the corresponding DFT spin density (see Figure 2). The (^3^LC-ppy)_min_ lies 2.79 eV above the (S_0_)_min_, which means that it is only 0.08 eV higher in energy than the ^3^MLCT equilibrium structure, the estimated emission energy (2.47 eV) being only 0.03 eV larger than that from (^3^MLCT)_min_ (2.44 eV). At the (^3^LC-ppy)_min_ geometry, the next state in energy at the TDDFT level is the ^3^MLCT, separated by 0.53 eV (Table S2). It is then plausible that radiative emission can also take place from the ^3^LC-ppy minimum since the population can there remain trapped, and the corresponding emission energy, being very similar to the ^3^MLCT one, agrees with the experimentally recorded emission (see Table 3).

**Table 3:** Theoretical and Experimental Data Characterizing the Emission of Complexes **1** and **2**: Emission Energy (*E*_em_), Phosphorescence Lifetime (τ), Emission Maxima (λ_max-em_), and the Emission Quantum Yield (Φ_em_)

Complex **1**					
theoretical data^a^			experimental data at RT^b^		
state	*E*_em_	τ	λ_max-em_	τ	Φ_em_
	(eV/nm)	(μs)	(nm/eV)	(μs)	
(^3^MLCT)_min_	2.05/605	3.23	595/2.08	0.565	0.196

Complex **2**					
theoretical data^a^			experimental data at RT^c^		
state	*E*_em_	τ	λ_max-em_	τ	Φ_em_
	(eV/nm)	(μs)	(nm/eV)	(μs)	
(^3^MLCT)_min_	2.44/508	2.80	524/2.37	1.25	0.71
(^3^LC-ppy)_min_	2.47/502	5.84			
(^3^LC-bpy)_min-td_	2.39/519	1.51			

a DFT B3LYP/(6-31G**+LANL2DZ)-PCM energies (dichloromethane for complex **1** and acetonitrile for complex **2**).

b Data measured in dichloromethane after excitation at 407 nm (3.05 eV).^6^

c Data measured in acetonitrile after excitation at 420 nm (2.95 eV).^9^

In order to study the population mechanism of the ^3^LC-ppy minimum, the TS state connecting the ^3^MLCT and ^3^LC-ppy minima, (^3^MLCT/^3^LC-ppy)_ts_, was characterized (see Figure S10). Due to convergence problems, the TS was obtained in the gas phase, and then, its energy was corrected accounting for the solvent. The (MLCT/LC-ppy)_ts_ is placed at 2.82 eV over the (S_0_)_min_, meaning that a small barrier of only 0.11 eV has to be overcome to reach the ^3^LC-ppy state from the (^3^MLCT)_min_. The phosphorescence lifetime of the ^3^LC-ppy state in its minimum was computed through TDDFT-SOC calculations. The three lowest singlet-triplet mixed states correspond to the ^3^LC-ppy sublevels (see Figure S13), and the next singlet-triplet mixed state is separated by 0.07 eV. The phosphorescence lifetime accounting for the three ^3^LC-ppy sublevels is equal to 5.84 μs, which, although slightly higher, is of the same order of magnitude than that characterizing the ^3^MLCT minimum (2.80 μs), further supporting a plausible involvement of such a state in the complex’s emission. Moreover, it must be noted that the computed DFT energy difference between the ^3^LC-ppy and ^3^MLCT minima (0.08 eV) is indeed too small to undoubtedly establish, within the employed level of theory, which of the two states corresponds to the absolute T_1_ minimum.

Regarding the ^3^LC-bpy state, its TDDFT-optimized structure also corresponds to the lowest T_1_ state at such a level of theory as stated above. The attempt of reoptimizing the geometry at the DFT level failed since the optimization converged to the ^3^MLCT minimum. This may be imputable to the similar nature that the two states partially share, the ^3^LC-bpy state being described, apart from a bpy-localized electronic promotion, by also an MLCT excitation. Its energy was then evaluated at the TDDFT minimum, performing a single-point DFT calculation. The spin density obtained from this calculation (Figure 2) confirms the predominant LC-bpy character of the state with 1.371 unpaired electrons on the dtb-bpy ligand compared with the 1.032e in the (^3^MLCT)_min_. The DFT energy of the ^3^LC-bpy state at the TDDFT (^3^LC-bpy)_min-td_ is equal to 2.78 eV. Therefore, the ^3^LC-bpy minimum is also energetically very close to the ^3^MLCT minimum, being only 0.07 eV above, and the estimated emission energy (2.39 eV) is similar to that from the ^3^MLCT minimum (2.44 eV). Consequently, the ^3^LC-bpy minimum could also contribute to the emission of complex **2**, a suggestion supported by the energy separation with the ^3^MLCT state (0.24 eV) computed at the TDDFT (^3^LC-bpy)_min-td_ (Table S2). The phosphorescence lifetime of the ^3^LC-bpy state has then been evaluated. Out of the corresponding TDDFT-SOC calculation (Figure S14), the three lower singlet-triplet mixed states are associated with the triplet ^3^MLCT state, the fourth state mainly corresponds to the singlet ^3^MLCT state, and states number 5, 6 and 7 describe the triplet ^3^LC-bpy state. Computing the corresponding phosphorescence lifetime using the data of the singlet-triplet mixed states 5, 6, and 7, a value of 1.51 μs is obtained, which consequently further supports the involvement of the ^3^LC-bpy state in the radiative process.

On the basis of these results, the ^3^MLCT, ^3^LC-ppy, and ^3^LC-bpy states are most likely involved in the emission of complex **2**. The so-described additional radiative decay path (*i.e.*, emission from the ^3^LC-ppy and ^3^LC-bpy minima) is represented in the left part of Figure 3b.

Further experimental insights into the emission process of complex **2** are obtained by analyzing the emission spectra at 300 and 77 K measured in acetonitrile and dichloromethane, respectively.^9^ Assuming that the change of the solvent does not significantly affect the emission properties, the comparison of the two spectra shows a sizable rigidochromic effect with a blueshift of 0.39 eV upon decreasing the temperature, the emission maxima being located at 524 (2.37) and 449 nm (2.76 eV) in the 300 and 77 K spectra, respectively. The rigidochromic effect is normally associated with an emission from an MLCT state, based on the assumption that an MLCT state will be more polar than the ground state, and a reorientation of the solvent therefore takes place when the system passes form the S_0_ to the ^3^MLCT state. At 77 K, such reorientation will be thermally blocked, preventing the stabilization of the excited state and therefore resulting in the increase in the emission energy registered in the rigidochromic effect. A clear difference in the dipole moment of the ^3^MLCT state with respect to the S_0_ state is indeed observable, being equal to around 2 and 13 D, respectively. It is then interesting to notice that the ground state is actually much more polar than the ^3^MLCT one. This would also lead to a rigidochromic blueshift at low temperatures and supports the participation of the ^3^MLCT state in the emission.

All the computed DFT relative energies for the characterized geometries of complexes **1** and **2** are reported in Table 4.

**Table 4:** DFT B3LYP/(6-31G**+LANL2DZ)-PCM Relative Energies (in eV) Computed for the S_0_ and Lowest-Energy T_1_ States at the Characterized Critical Points of Complexes **1** and **2**^a^

Complex **1**		
	S_0_	T_1_
(S_0_)_min_	0.00	2.59
(^3^MLCT)_min_	0.27	2.32
(^3^MLCT/^3^MC)_ts_	0.91	3.06
(^3^MC)_min_	1.63	2.94
(^3^MC/S_0_)_stc-mecp_	3.16	3.18

Complex **2**		
	S_0_	T_1_
(S_0_)_min_	0.00	2.97
(^3^MLCT)_min_	0.27	2.71
(^3^MLCT/^3^MC)_ts_	0.79	3.27
(^3^MC)_min_	1.54	3.08
(^3^MC/S_0_)_stc_	3.47	3.48
(^3^LC-ppy)_min_	0.32	2.79
(^3^MLCT/^3^LC-ppy)_ts_	0.26	2.82
(^3^LC-bpy)_min-td_	0.39	2.78

a All reported energies are referred with respect to the S_0_ energy at the (S_0_)_min_ of the corresponding complex.

Up to now, we have adopted the usual model accordingly to which in transition-metal complexes, the presence of considerable spin–orbit coupling leads to the population of the lowest triplet state. However, it is interesting to check the validity of such an assumption to see if any significant difference appears between the two complexes. The lowest singlet excited states were then computed at the TDDFT level at the optimized (S_0_)_min_ geometries. Considering the excitation wavelength employed in the experimental measurements of the emission quantum yield, equal to 407 and 420 nm (3.05 and 2.95 eV) for complexes **1** and **2**,^6,9^ respectively, the initially populated excited singlet state appears to be in both cases an LC-ppy state, hereafter ^1^LC-ppy, placed at 3.11 and 3.33 eV and having oscillator strength values for the transition from the ground state of 0.063 and 0.053, respectively. The evolution of such a state was studied by optimizing its minimum, hereafter (^1^LC-ppy)_min-td_. For both complexes, at the (^1^LC-ppy)_min-td_ structure, the ^1^MLCT state is still lower in energy than the ^1^LC-ppy state, and it is therefore reasonable to assume that the ^1^MLCT state will be populated and that the system will then evolve to its minimum, (^1^MLCT)_min-td_. The ^1^MLCT and ^3^MLCT states are very close in energy in all the computed geometries, and the (^1^MLCT)_min-td_ is no exception. In fact, the (^1^MLCT)_min-td_ and (^3^MLCT)_min_ minima are indeed almost indistinguishable, both geometrically and energetically. On the basis of these static results, we can then conclude that in both complexes, the initially excited population will mainly reach the (^3^MLCT)_min_ and that the initial decay does not appear to be substantially different in the two molecules. All the energies characterizing this path are reported in Table S3.

Finally, some comments on the choice of functional here adopted (the global hybrid B3LYP functional) are in order. It is generally accepted that B3LYP tends to underestimate charge-transfer states.^39^ Although the choice of B3LYP has been motivated by its ability of providing emission energies and geometries for complex **1** in agreement with experimental data (see refs (6) and (9)), since our conclusions depends on the relative energies of MLCT, LC, and MC states, we decided to evaluate the influence of the adopted functional on the presented results.

Mostly motivated by the above mentioned pitfall of B3LYP, Felix Plasser and Andreas Dreuw published a work in which the excited states of the Ir(ppy)_3_ complex were characterized using the latter functional and the range-separated wB97 one, obtaining a very different picture accordingly to the two functionals.^40^ For these reasons, we decided to evaluate the performance of wB97 for the problem at hand, using as a testing ground the description of the nonradiative ^3^MC decay path. The S_0_, ^3^MLCT, and ^3^MC states have been then reoptimized with such a functional and also, in order to have a more global vision, with the following ones: PBE, BLYP, and BP86 (GGA functionals); PBE0 (hybrid functional); M06 (meta-hybrid functional); CAM-B3LYP (range-separated functionals). The obtained results are presented in Table S4. As it is possible to observe, GGA functionals provide higher energy differences between the ^3^MLCT minimum and the ^3^MC minimum than B3LYP, while on the contrary, M06 and range-separated functionals provide a smaller energy difference between the two minima. Those differences are more pronounced for complex **2**, where according to the wB97 results, the ^3^MC minimum is placed 0.09 eV below the ^3^MLCT one. Considering also that at the wB97 level, the S_0_-^3^MC gap at the (^3^MC)_min_ is significantly reduced (0.53 eV), those results will point to a very favorable nonradiative decay through the ^3^MC state, which is in disagreement with the high experimental emission quantum yield of complex **2**. A similar picture emerges also from CAM-B3LYP and M06 energies. Based on such a result, we considered that the wB97, CAM-B3LYP, and M06 functionals are not suited for the description of complex **2**, and consequently, we refrain from using them. A problematic performance of the range-separated functional as CAM-B3LYP for the description of the excited states of transition-metal complexes was also pointed out in a benchmark study of Barone and co-workers, who tested the performance of various functionals for the simulation of absorption spectra for some Ir(III) and Pt(II) complexes and concluded that “CAM-B3LYP has a net trend to overestimate the vertical excitation energies with respect to the experimental values”.^41^ On the other hand, another benchmark study, authored by Atkins and co-workers, concluded studying three Ru(II) complexes that hybrid functionals, as B3LYP, provide better excitation energies while pure functionals provide, typically, better energy gaps (both quantities evaluated with respect to MS-CASPT2 results).^42^

As a further comparison, in Table S5, we collected the vertical emission energy obtained with the different functionals. As observed in Table S5, the vertical emission energy calculated with other functionals supports the values obtained with B3LYP, the maximum difference being within 0.2 eV.

On the basis of those considerations and results, we then consider more than reasonable the use of the B3LYP functional for the description of the here studied two complexes.

## Conclusions

The main radiative and nonradiative decays experimented by the [Ir(ppy)_2_(bpy)]^+^ and [Ir(diFppy)_2_(dtb-bpy)]^+^ cyclometalated Ir(III) complexes (complexes **1** and **2**, respectively) have been theoretically investigated by performing DFT-based calculations. Despite their similar chemical structures, complex **2** is characterized by a much higher emission quantum yield (0.71) than complex **1** (0.196). The reason for such a remarkable difference does not appear to be related to a different participation in the systems’ photophysics of the nonradiative decay path mediated by a metal-centered ^3^MC state since comparable energy barriers characterize such a decay in the two complexes. The difference in the emission quantum yield neither appears to be associated with a different emission efficiency from the emitting ^3^MLCT state since similar phosphorescence lifetimes are computed from the ^3^MLCT minima of both complexes. The only plausible cause that has been identified here is the presence of two additional emissive T_1_ minima characterized for complex **2** but not in complex **1**. The states associated with such minima are indeed present in both complexes, but while in complex **2**, their equilibrium structures correspond to minima of the lowest-energy triplet T_1_, in complex **1**, the states can always decay to the lower ^3^MLCT state. The involvement of these two minima, associated respectively to ligand-centered ^3^LC-ppy and ^3^LC-bpy states, is supported by their emission energies, which are comparable to the emission wavelength experimentally recorded, and by the values estimated for the phosphorescence lifetime, which are similar to that characterizing the ^3^MLCT state.

In conclusion, for complex **2**, the presence of two additional LC-type triplet minima, in addition to that associated to the usual ^3^MLCT state, appears as a key factor leading to its remarkable emission quantum yield. The ^3^LC states may increase the emission efficiency of the complex either by emitting themselves and/or by providing additional routes in the system that prevent the decay along the nonradiative ^3^MC pathway. Complex **2** therefore constitutes a noticeable example in which the presence of low-lying ^3^LC states, normally believed to be unfavorable for the complex’s emission, is indeed the key for an efficient photoluminescence.

Finally, a consideration regarding molecular design criteria for achieving high emission yields is in order. It is somehow spread in the literature of iTMCs for electroluminescent applications that the price that one has to pay in order to chemically blueshift the emission of a complex is a loss in its emission efficiency, associated to the fact that higher emitting states implicate easier access to nonradiative ^3^MC states. Although the higher accessibility of ^3^MC states is confirmed here (the energy gap between the ^3^MLCT and ^3^MC minima being 0.25 eV smaller for the blueshifted complex **2** than for complex **1**), it appears that the blueshift of the original emitting ^3^MLCT state is the key for the high emission of complex **2** since it is the destabilization of the ^3^MLCT state that causes other states (the ^3^LC-ppy and ^3^LC-bpy states in this case) to display emitting T_1_ minima contributing to the total emission of the system.

## Supporting Information Available

Analysis of the optimized geometries, molecular orbitals, imaginary frequencies at the obtained TS, TDDFT-SOC states for the evaluation of the radiative lifetimes, energies of the relevant excited states at all characterized critical points, electronic energies using different functionals, and Cartesian coordinates of all structures (PDF)
