# Supplementary material for: On the Importance of Ligand-Centered Excited States in the Emission of Cyclometalated Ir(III) Complexes
Source: Inorg Chem. 2021 Aug 16;60(17):13222–32. doi: 10.1021/acs.inorgchem.1c01604 (PMC8424641; doi:10.1021/acs.inorgchem.1c01604)
Supplement: Supplementary file 1 — ic1c01604_si_001.pdf [file ic1c01604_si_001.pdf]

Supplementary Information

On the Importance of Ligand-Centered Excited States in the Emission of Cyclometalated Ir(III) Complexes

Iván Soriano-Díaz, Enrique Ortí,* and Angelo Giussani*

Instituto de Ciencia Molecular, Universidad de Valencia, Catedrático José Beltrán 2,
46980 Paterna, Spain. E-mail: enrique.orti@uv.es, angelo.giussani@uv.es

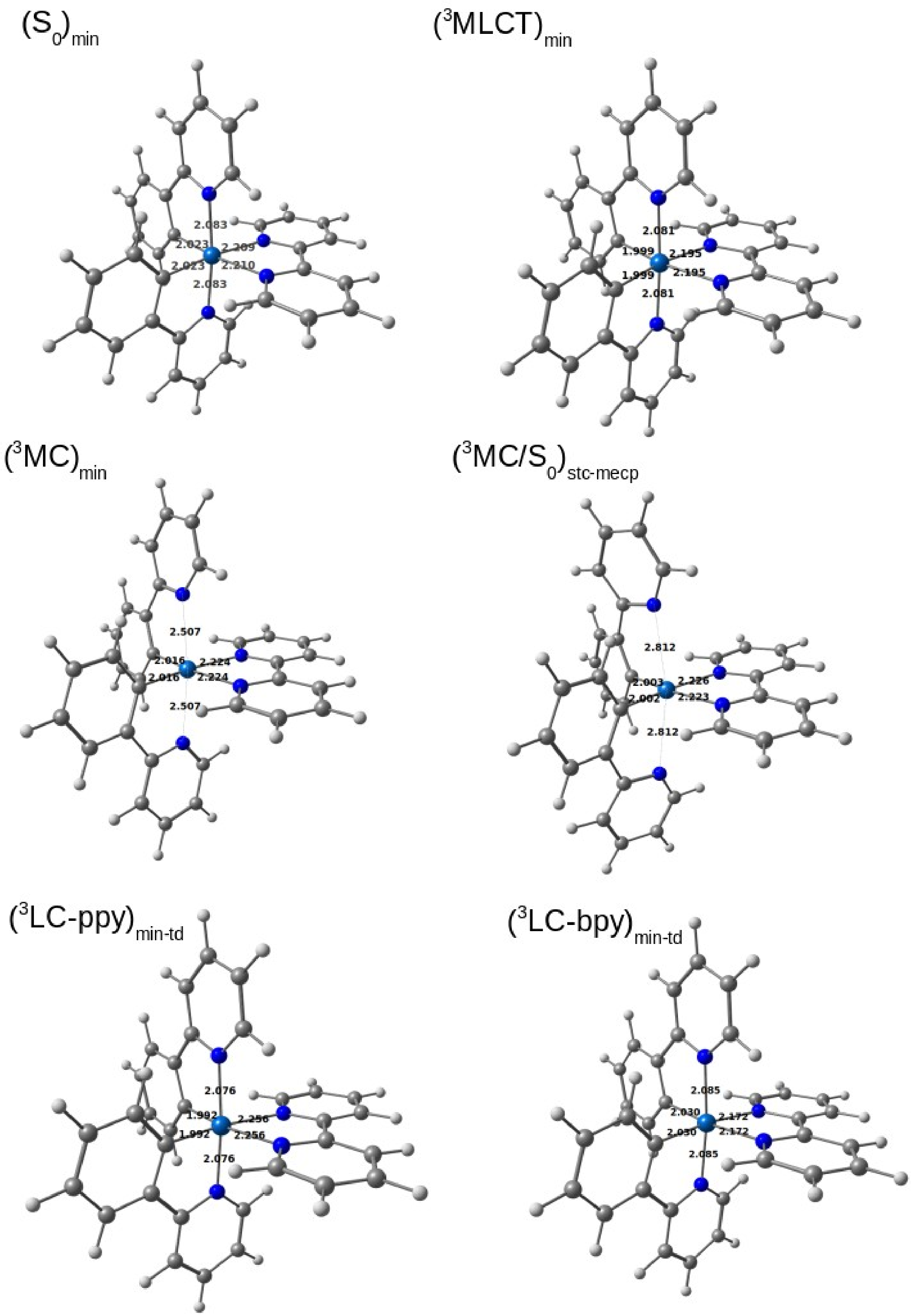

Figure S1. Comparison of the bond lengths (in Å) characterizing the Ir coordination in the B3LYP/(6-31G**+LANL2DZ)-optimized geometries of complex 1

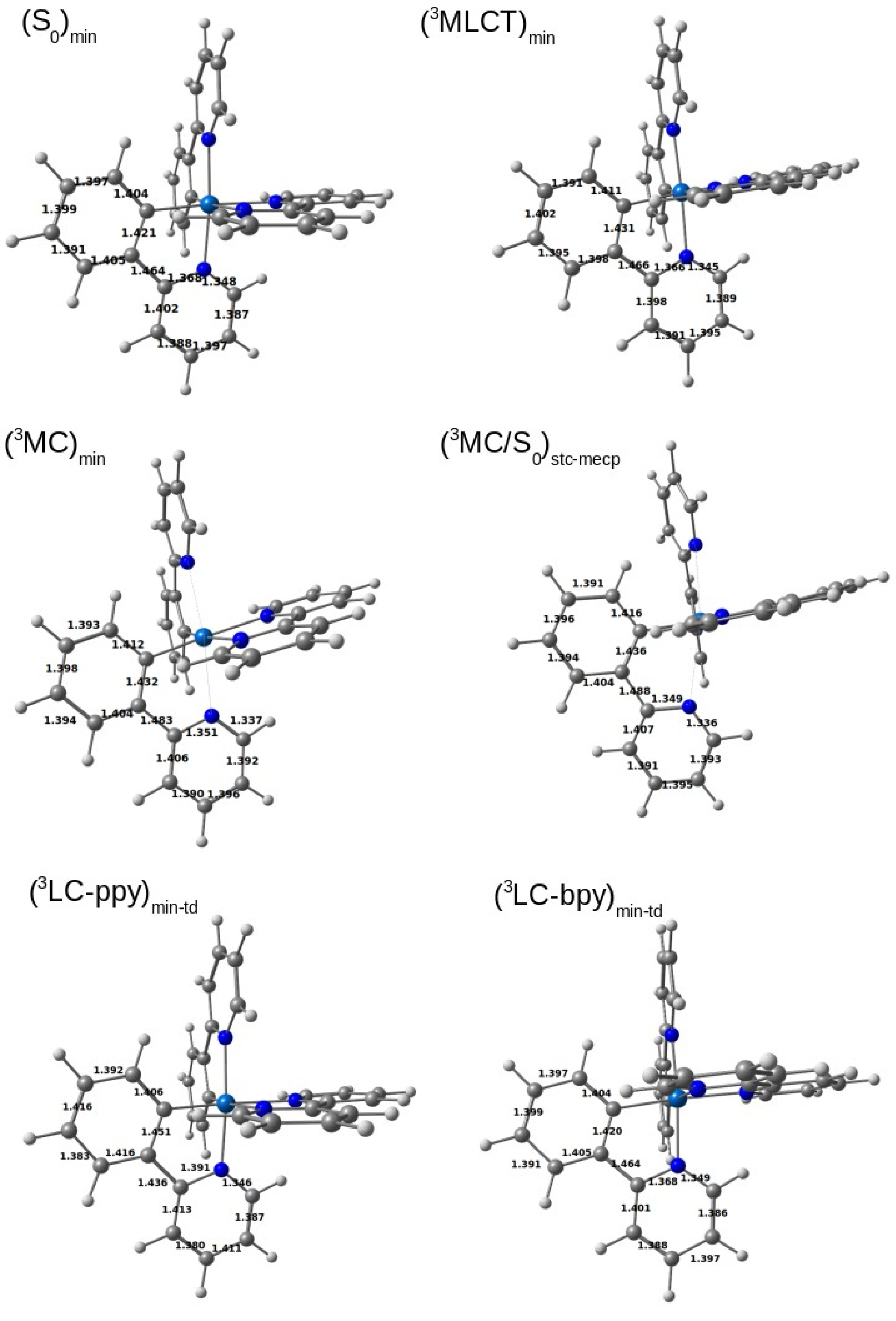

Figure S2. Comparison of the bond lengths (in Å) characterizing the ppy ligand in the B3LYP/(6-31G**+LANL2DZ)-optimized geometries of complex **1**

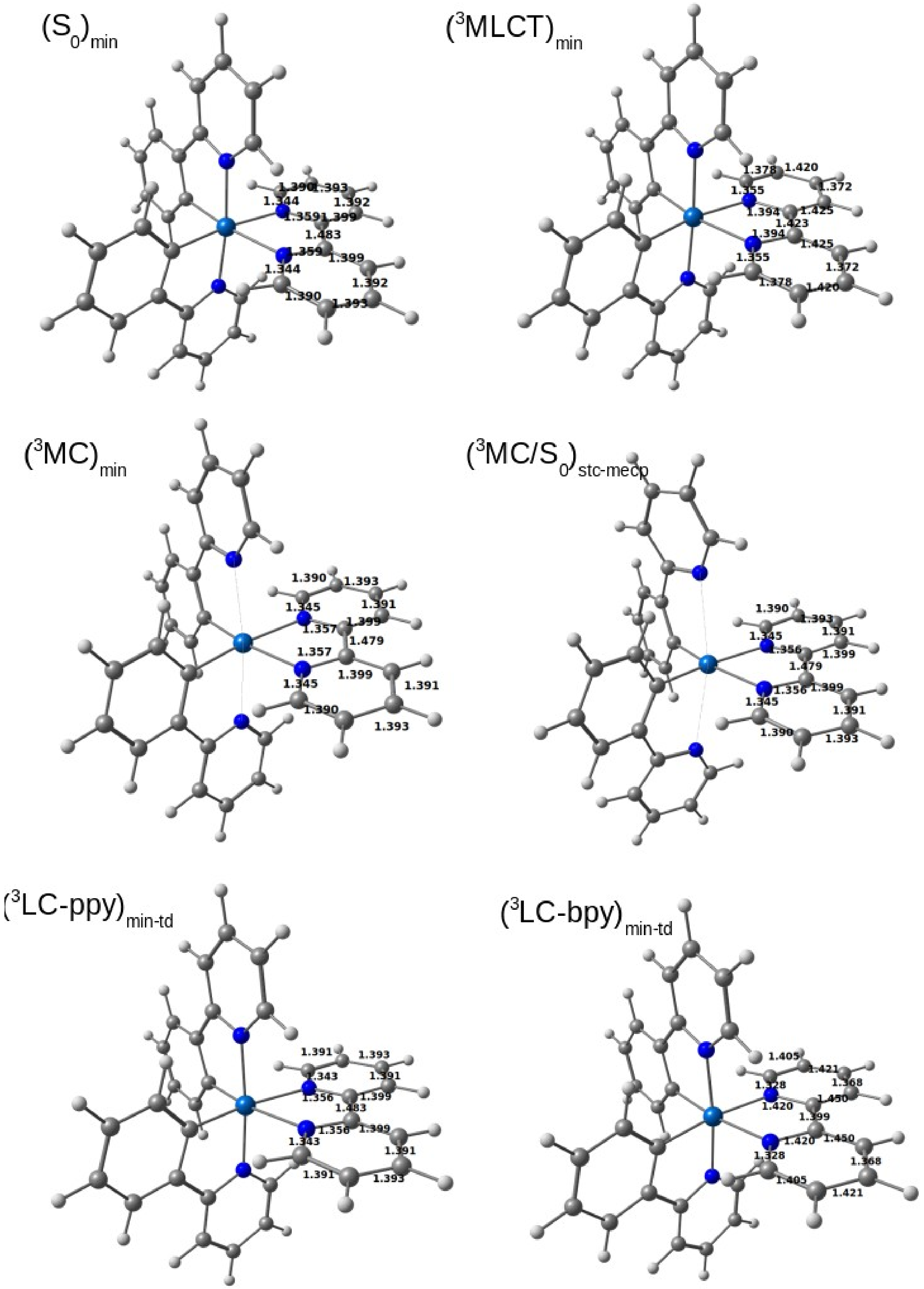

Figure S3. Comparison of the bond lengths (in Å) characterizing the bpy ligand in the B3LYP/(6-31G**+LANL2DZ)-optimized geometries of complex **1**

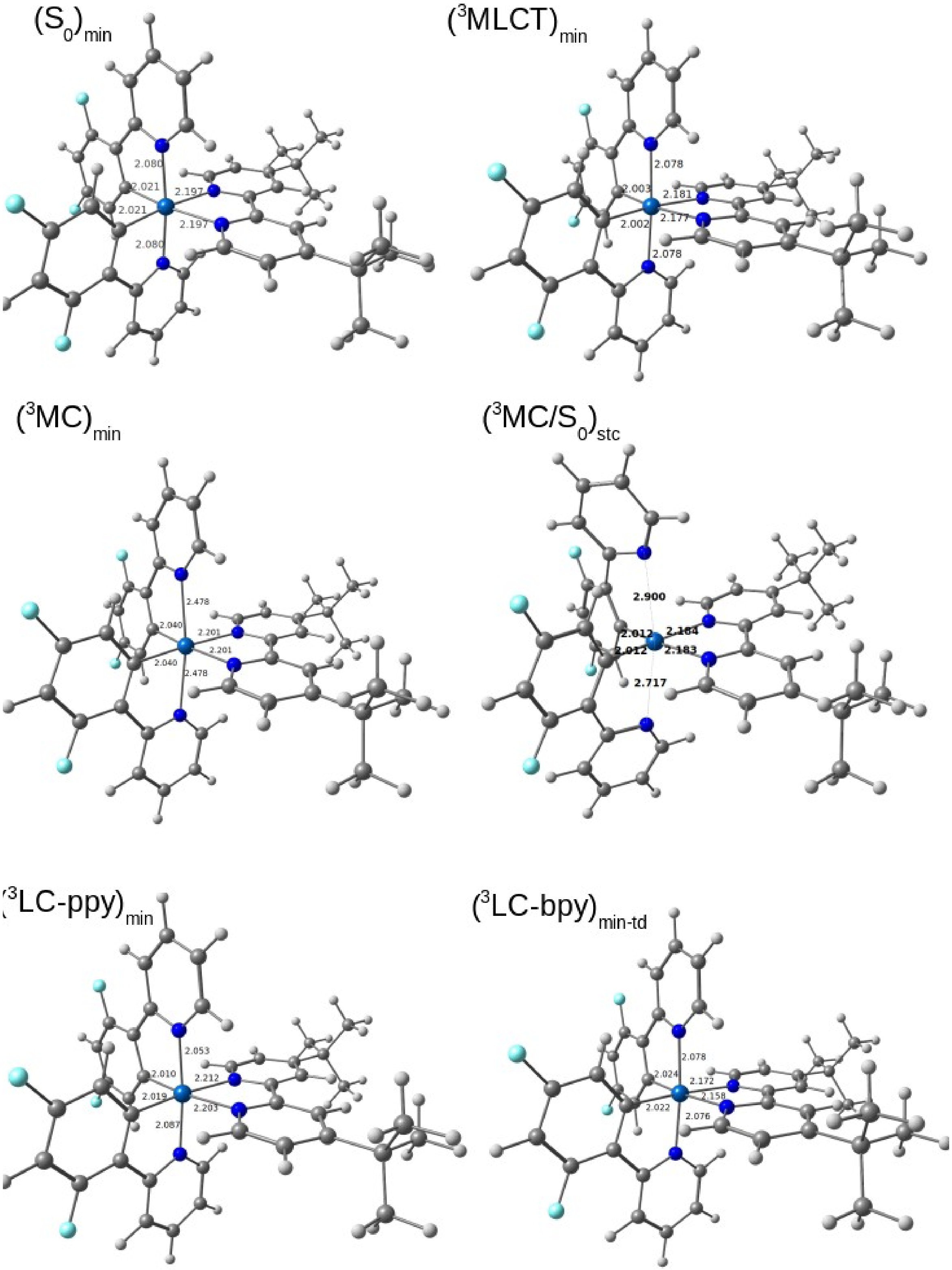

Figure S4. Comparison of the bond lengths (in Å) characterizing the Ir coordination in the B3LYP/(6-31G**+LANL2DZ)-optimized geometries of complex **2**

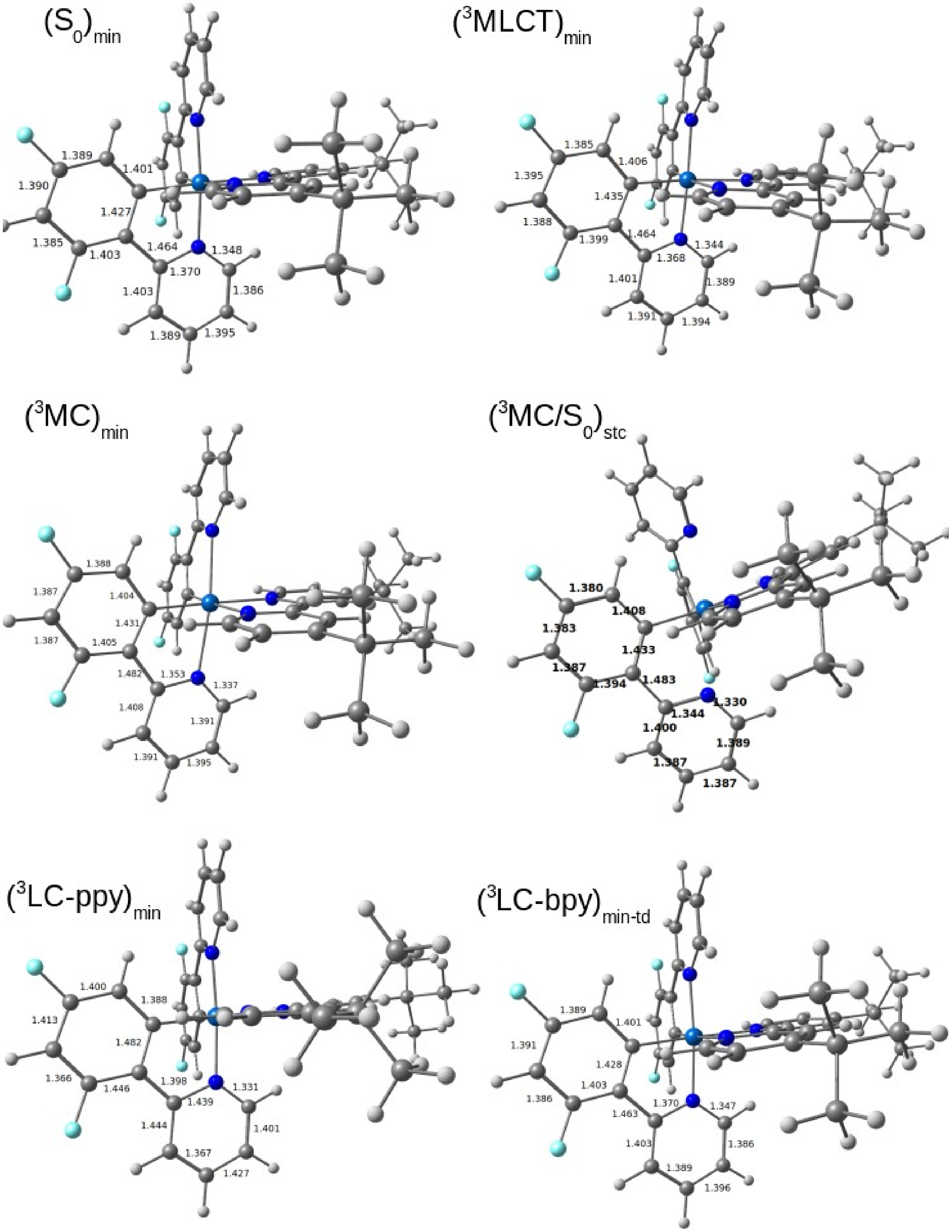

Figure S5. Comparison of the bond lengths (in Å) characterizing the ppy ligand in the B3LYP/(6-31G**+LANL2DZ)-optimized geometries of complex **2**

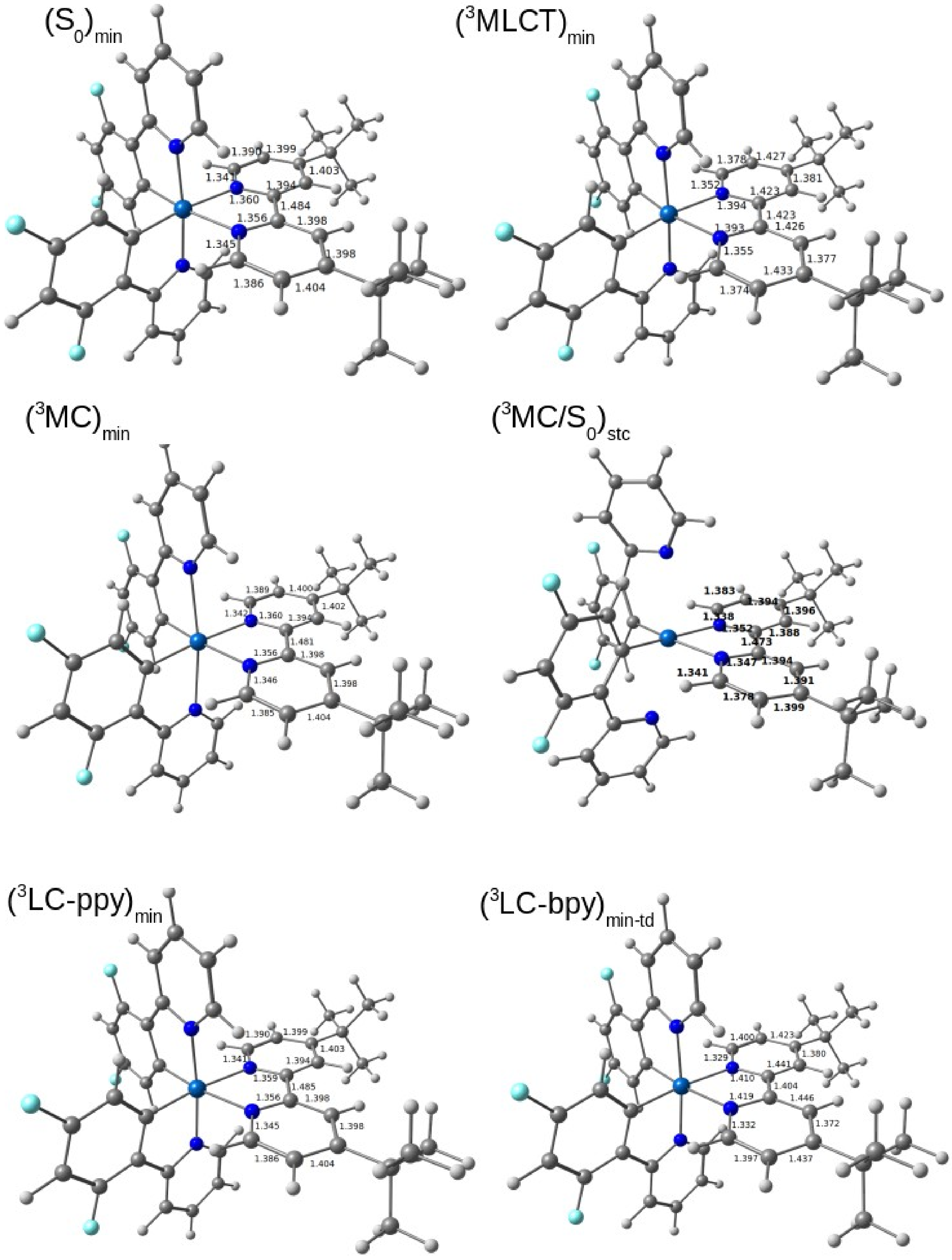

Figure S6. Comparison of the bond lengths (in Å) characterizing the bpy ligand in the B3LYP/(6-31G**+LANL2DZ)-optimized geometries of complex **2**

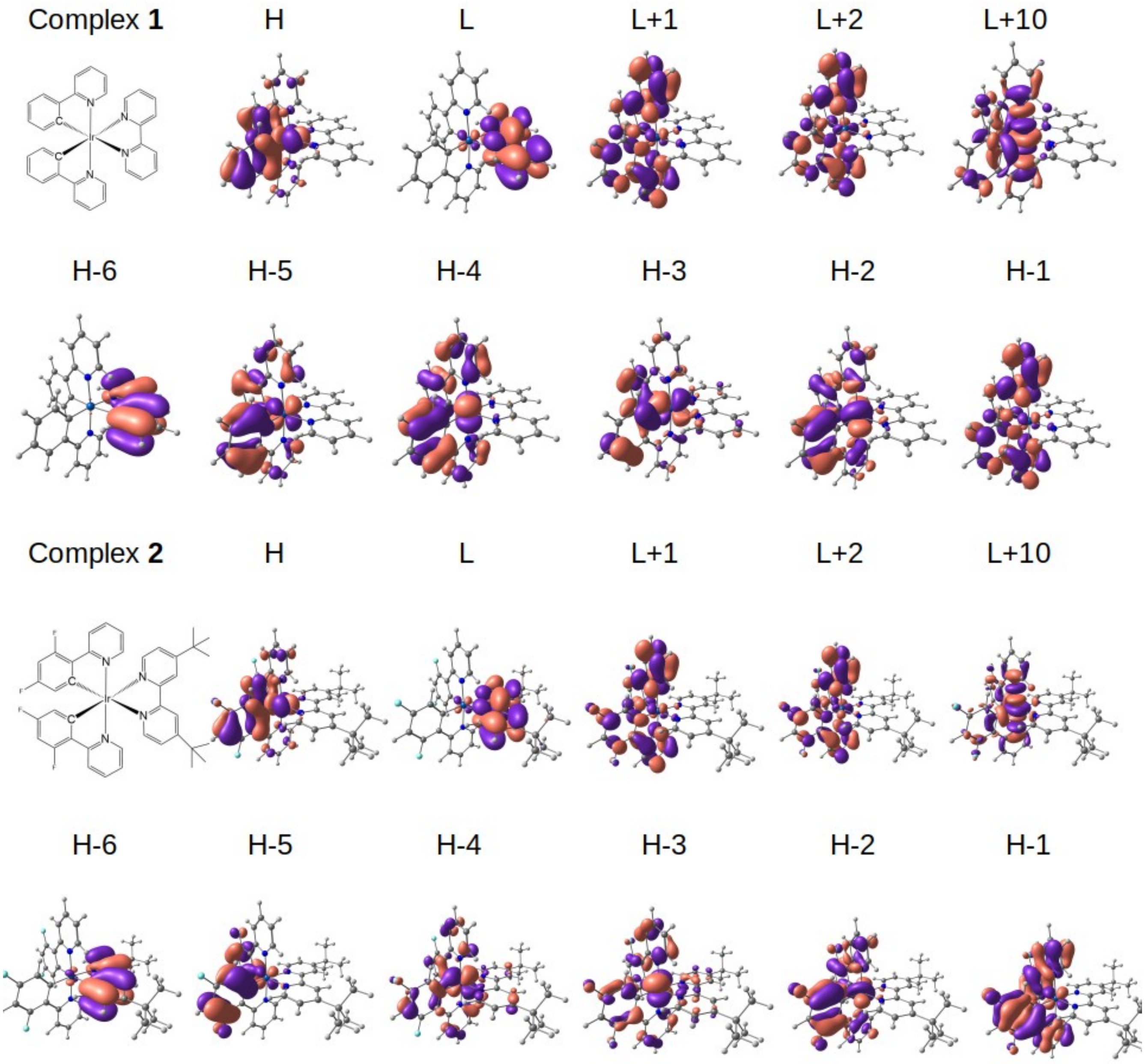

Figure S7. Isovalue contour plots (±0.03 a.u.) of molecular orbitals of complex 1 and 2 computed at the DFT B3LYP/(6-31G**+LANL2DZ) PCM level. H and L denote the HOMO and LUMO, respectively. The structure of the two complexes is also reported.

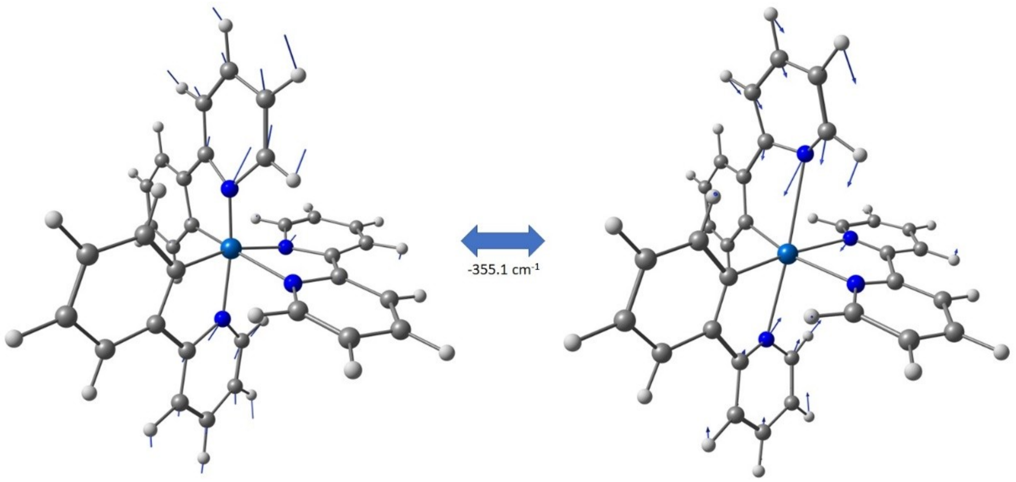

Figure S8. Imaginary frequency characterizing the (^{3}MLCT/^{3}MC)$_{ts}$ transition state in complex **1**.

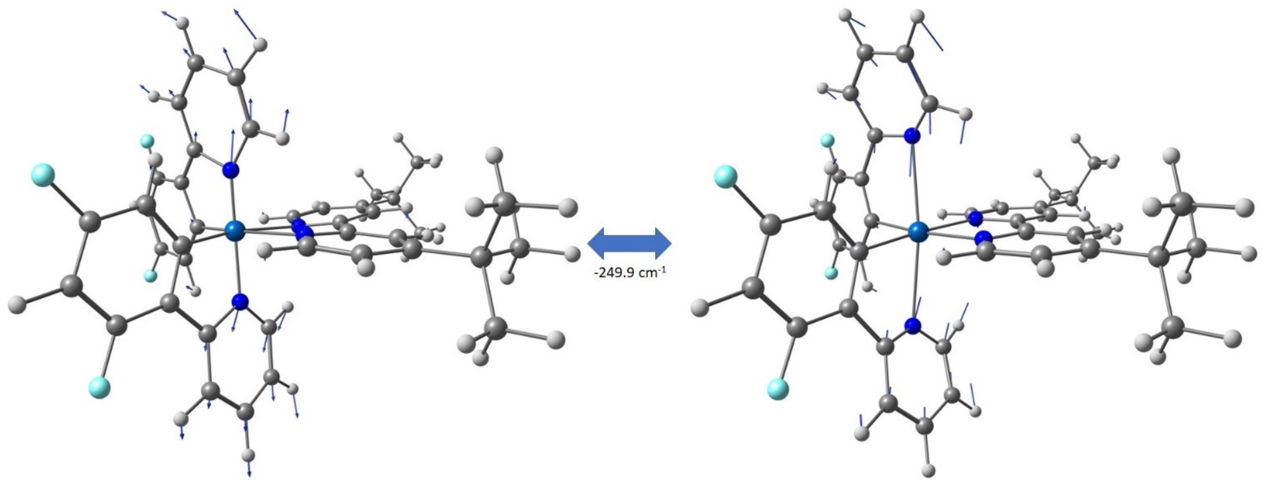

Figure S9. Imaginary frequency characterizing the (^{3}MLCT/^{3}MC)$_{ts}$ transition state in complex **2**.

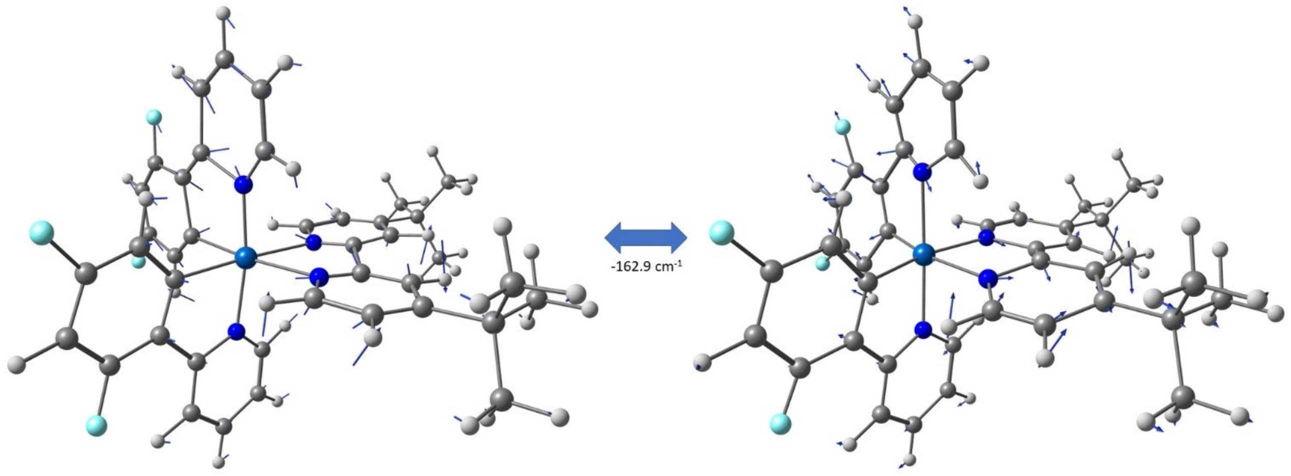

Figure S10. Imaginary frequency characterizing the (^{3}MLCT/^{3}LC-ppy)$_{ts}$ transition state in complex **2**.

	E(cm-1)	Weight	Real	Imag	: Root	Spin	Ms
STATE 0:	0.00						
		0.99381	0.00091	-0.99690	: 0	0	0
STATE 1:	11540.41						
		0.60102	-0.77525	-0.00210	: 1	1	0
		0.16294	0.40365	0.00081	: 1	1	-1
		0.01925	0.00039	-0.13875	: 3	1	-1
		0.16294	-0.40365	-0.00138	: 1	1	1
		0.01925	0.00037	-0.13875	: 3	1	1
STATE 2:	11556.36						
		0.02149	-0.14661	-0.00050	: 2	0	0
		0.02586	0.00055	-0.16080	: 3	1	0
		0.46559	-0.68234	-0.00208	: 1	1	-1
		0.46559	-0.68233	-0.00259	: 1	1	1
STATE 3:	11672.96						
		0.01410	-0.00049	0.11875	: 4	0	0
		0.33431	-0.57819	-0.00240	: 1	1	0
		0.01368	0.11696	0.00049	: 2	1	0
		0.30764	-0.55464	-0.00219	: 1	1	-1
		0.30764	0.55464	0.00242	: 1	1	1

Figure S11 TDDFT-SOC lowest singlet-triplet mixed states of complex **1** computed at the ^{3}MLCT minimum

	E(cm-1)	Weight	Real	Imag	: Root	Spin	Ms
STATE 0:	0.00						
		0.99535	-0.09088	0.99353	: 0	0	0
STATE 1:	14855.62						
		0.88644	0.89767	0.28395	: 1	1	0
		0.01914	0.01295	-0.13773	: 2	1	-1
		0.02761	0.16349	0.02973	: 3	1	-1
		0.01914	0.06861	-0.12013	: 2	1	1
		0.02761	-0.15085	-0.06970	: 3	1	1
STATE 2:	14881.30						
		0.01548	0.12437	0.00281	: 1	0	0
		0.02849	0.16876	0.00382	: 2	0	0
		0.05306	0.00521	-0.23029	: 3	1	0
		0.44161	-0.02803	0.66395	: 1	1	-1
		0.44161	0.00203	-0.66454	: 1	1	1
STATE 3:	15003.51						
		0.02146	-0.00642	-0.14634	: 5	0	0
		0.03297	0.18140	-0.00796	: 2	1	0
		0.45805	0.02322	0.67640	: 1	1	-1
		0.45805	0.03608	0.67583	: 1	1	1

Figure S12 TDDFT-SOC lowest singlet-triplet mixed states of complex **2** computed at the ^{3}MLCT minimum

	E(cm-1)	Weight	Real	Imag	: Root	Spin	Ms
STATE 0:	0.00						
		0.99552	0.98802	0.13907	: 0	0	0
STATE 1:	18109.98						
		0.39144	-0.54121	-0.31390	: 1	1	0
		0.29536	-0.48038	0.25414	: 1	1	-1
		0.29536	0.01794	0.54317	: 1	1	1
STATE 2:	18111.63						
		0.54990	0.72809	-0.14064	: 1	1	0
		0.21527	-0.32385	0.33226	: 1	1	-1
		0.21527	0.42429	0.18775	: 1	1	1
STATE 3:	18122.53						
		0.04062	0.12163	-0.16071	: 1	1	0
		0.46985	0.67222	-0.13406	: 1	1	-1
		0.46985	0.05355	0.68336	: 1	1	1

Figure S13 TDDFT-SOC lowest singlet-triplet mixed states of complex **2** computed at the ^{3}LC-ppy minimum

	E(cm-1)	Weight	Real	Imag	: Root	Spin	Ms
STATE 0:	0.00						
		0.99587	-0.60434	0.79413	: 0	0	0
STATE 1:	14756.26						
		0.81004	-0.02006	0.89980	: 1	1	0
		0.01378	-0.10981	0.04150	: 1	1	-1
		0.02826	0.16449	-0.03463	: 2	1	-1
		0.03052	0.02362	0.17309	: 3	1	-1
		0.01378	-0.10786	-0.04635	: 1	1	1
		0.02826	0.16278	0.04193	: 2	1	1
		0.03052	0.03131	-0.17187	: 3	1	1
STATE 2:	14772.61						
		0.09347	0.29418	-0.08324	: 1	0	0
		0.03776	-0.18698	0.05291	: 2	0	0
		0.04889	0.06020	0.21277	: 3	1	0
		0.38725	-0.20197	-0.58861	: 1	1	-1
		0.38725	0.13638	0.60717	: 1	1	1
STATE 3:	14911.41						
		0.02869	-0.01349	0.16885	: 1	0	0
		0.02248	-0.01194	0.14946	: 5	0	0
		0.02506	0.15780	0.01261	: 1	1	0
		0.05254	-0.22848	-0.01826	: 2	1	0
		0.01508	-0.12240	-0.00978	: 4	1	0
		0.41203	-0.01464	-0.64173	: 1	1	-1
		0.41203	0.11637	-0.63126	: 1	1	1
STATE 4:	15151.24						
		0.68050	-0.60402	-0.56184	: 1	0	0
		0.01189	-0.07426	0.07984	: 3	1	0
		0.06122	0.03704	-0.24464	: 1	1	-1
		0.03586	0.09142	-0.16585	: 2	1	-1
		0.02889	0.13957	0.09700	: 3	1	-1
		0.06122	-0.24133	0.05462	: 1	1	1
		0.03586	-0.15881	0.10316	: 2	1	1
		0.02889	0.10684	0.13219	: 3	1	1
STATE 5:	17993.18						
		0.08540	0.28782	-0.05058	: 1	1	0
		0.01219	-0.10873	0.01911	: 2	1	0
		0.43533	0.24185	0.61387	: 2	1	-1
		0.01077	-0.09490	0.04197	: 3	1	-1
		0.43533	-0.01808	0.65954	: 2	1	1
		0.01077	0.10352	0.00710	: 3	1	1
STATE 6:	18016.51						
		0.02376	-0.06691	0.13885	: 1	0	0
		0.69885	0.75309	0.36292	: 2	1	0
		0.02677	0.01015	-0.16330	: 1	1	-1
		0.10194	0.28326	0.14734	: 2	1	-1
		0.02677	0.12140	-0.10968	: 1	1	1
		0.10194	-0.29174	-0.12975	: 2	1	1
STATE 7:	18017.44						
		0.07951	0.27744	0.05035	: 1	0	0
		0.20489	-0.08083	0.44537	: 2	1	0
		0.33468	-0.05201	-0.57617	: 2	1	-1
		0.33468	-0.25115	0.52115	: 2	1	1

Figure S14 TDDFT-SOC lowest singlet-triplet mixed states of complex **2** computed at the ^{3}LC-bpy minimum.

Table S1. TDDFT B3LYP/(ZORA-DEF2-TZVP+SARC-ZORA-TZVP) energies computed with Orca at the S_0 minimum and at the minima for which the radiative lifetime was evaluated for complexes **1** and **2**.[a] In parenthesis, the difference with respect to the corresponding value computed with Gaussian at the TDDFT B3LYP/(6-31G**+LANL2DZ) level is reported.

complex **1**						
	triplets					singlet
	S_0	^{3}MLCT	^{3}MC	^{3}LC-ppy	^{3}LC-bpy	S_1
$(S_0)_{min}$	0.00	1.98 (-0.05)	-	-	-	2.01 (-0.04)
$(^3MLCT)_{min}$	0.29 (0.01)	1.77 (-0.02)	-	-	-	1.80 (-0.02)
complex **2**						
	triplets					singlet
	S_0	^{3}MLCT	^{3}MC	^{3}LC-ppy	^{3}LC-bpy	S_1
$(S_0)_{min}$	0.00	2.41 (-0.04)	-	-	-	2.44 (-0.04)
$(^3MLCT)_{min}$	0.29 (0.01)	2.19 (-0.02)	-	-	-	2.22 (-0.02)
$(^3LC\text{-}ppy)_{min\text{-}td}$	0.32 (0.02)	2.69 (-0.02)	-	2.57 (-0.02)	-	2.72 (-0.01)
$(^3LC\text{-}bpy)_{min\text{-}td}$	0.41 (0.01)	2.32 (-0.02)	-	-	2.62 (-0.03)	2.37 (-0.02)

[a] All reported energies are referred with respect to the S_0 energy at the $(S_0)_{min}$ of the corresponding complex

Table S2. TDDFT B3LYP/(6-31G**+LANL2DZ) PCM energies (eV) computed with Gaussian for complexes **1** and **2** at the optimized geometries of all the characterized minima.[a]

complex **1**						
	triplets					singlets
	S_0	^{3}MLCT	^{3}MC	^{3}LC-ppy	^{3}LC-bpy	^{1}MLCT
$(S_0)_{min}$	0.00	2.43				2.46
$(^3MLCT)_{min}$[b]	0.27	2.18				2.21
$(^3MC)_{min}$[b]	1.62	4.05	2.81			4.09
$(^3LC\text{-}ppy)_{min\text{-}td}$[c]	0.18	2.51		2.58		2.54
$(^3LC\text{-}bpy)_{min\text{-}td}$[c]	0.41	2.38			2.57	2.41
$(S_1)_{min}$[c]	0.25	2.18				2.21
complex **2**						
	triplets					singlets
	S_0	^{3}MLCT	^{3}MC	^{3}LC-ppy	^{3}LC-bpy	^{1}MLCT
$(S_0)_{min}$	0.00	2.85				2.89
$(^3MLCT)_{min}$[b]	0.27	2.60				2.65
$(^3MC)_{min}$[b]	1.54	4.45	2.98			4.50
$(^3LC\text{-}ppy)_{min\text{-}td}$[c]	0.32	3.13		2.60		3.15
$(^3LC\text{-}bpy)_{min\text{-}td}$[c]	0.39	2.83			2.59	2.79
$(S_1)_{min}$[c]	0.25	2.61				2.64

[a] All reported energies are referred with respect to the S_0 energy at the $(S_0)_{min}$ of the corresponding complex.
[b] Optimized at the DFT B3LYP/(6-31G**+LANL2DZ) PCM level.
[c] Optimized at the TDDFT B3LYP/(6-31G**+LANL2DZ) PCM level.

Table S3. TDDFT B3LYP/(6-31G**+LANL2DZ) PCM energies (eV) computed with Gaussian for complexes **1** and **2** at the geometries relevant for the initial decay to the $(^3MLCT)_{min}$.[a]

complex **1**					
	S_0	^{3}MLCT	^{1}MLCT	^{1}LC-ppy	^{3}LC-ppy
$(S_0)_{min}$	0.00	2.43	2.46	3.11[b]	2.75
$(^1LC\text{-}ppy)_{min\text{-}td}$	0.22	2.51	2.54	2.91	2.61
$(^1MLCT)_{min\text{-}td}$	0.25	2.18	2.21	-	-
complex **2**					
	S_0	^{3}MLCT	^{1}MLCT	^{1}LC-ppy	^{3}LC-ppy
$(S_0)_{min}$	0.00	2.85	2.89	3.33[c]	2.89
$(^1LC\text{-}ppy)_{min\text{-}td}$	0.23	2.96	2.99	3.13	2.79
$(^1MLCT)_{min\text{-}td}$	0.25	2.61	2.64	-	-

[a] All reported energies are referred with respect to the S_0 energy at the $(S_0)_{min}$ of the corresponding complex
[b] Oscillator strength for the S0 -> ^{1}LC-ppy transition equal to 0.0629
[c] Oscillator strength for the S0 -> ^{1}LC-ppy transition equal to 0.0528

Table S4. DFT/(6-31G**+LANL2DZ) PCM relative energies (in eV) computed with different functionals for different states and geometries of complexes **1** and **2**.[a] The relative differences with respect to the value obtained using B3LYP are reported within parentheses.

complex **1**				
Functional		^{3}MLCT at $(S_0)_{min}$	^{3}MLCT at $(^3MLCT)_{min}$	^{3}MC at $(^3MC)_{min}$
GGA	PBE	0.27 (0.01)	0.00	0.84 (0.22)
	BLYP	0.14 (–0.12)	0.00	0.71 (0.09)
	BP86	0.15 (–0.11)	0.00	–
hybrid	PBE0	0.28 (+0.02)	0.00	–
	**B3LYP**	**0.26**	**0.00**	**0.62**
meta-hybrid	M06	0.29 (+0.03)	0.00	0.59 (–0.03)
range-separated	CAM-B3LYP	0.33 (+0.09)	0.00	0.58 (–0.04)
	wB97	0.39 (+0.13)	0.00	0.37 (–0.25)
complex **2**				
Functional		^{3}MLCT at $(S_0)_{min}$	^{3}MLCT at $(^3MLCT)_{min}$	^{3}MC at $(^3MC)_{min}$
GGA	PBE	0.27 (0.01)	0.00	0.41 (0.04)
	BLYP	0.13 (–0.13)	0.00	0.47 (0.10)
	BP86	0.14 (–0.12)	0.00	0.66 (0.29)
hybrid	**B3LYP**	**0.26**	**0.00**	**0.37**
meta-hybrid	M06	0.28 (0.02)	0.00	0.14 (–0.23)
range-separated	CAM-B3LYP	0.33 (0.07)	0.00	0.14 (–0.23)
	wB97	0.39 (0.13)	0.00	–0.09 (–0.46)

[a] For each functional, the energy of the ^{3}MLCT state at $(^3MLCT)_{min}$ geometry is taken as zero energy.

Table S5. DFT/(6-31G**+LANL2DZ) PCM vertical emission energies (in eV) from the ^{3}MLCT state computed with different functionals for complexes **1** and **2**. The relative differences with respect to the value obtained using B3LYP are reported within parentheses.

Functional		complex **1**[a]	complex **2**[b]
GGA	PBE	1.90 (–0.15)	2.46 (0.02)
	BLYP	2.17 (0.12)	2.23 (-0.21)
	BP86	2.07 (0.02)	2.23 (-0.21)
hybrid	**B3LYP**	**2.05**	**2.44**
meta-hybrid	M06	2.04 (–0.01)	2.42 (0.02)
range-separated	CAM-B3LYP	2.17 (0.12)	2.59 (0.15)
	wB97	2.23 (0.18)	2.64 (0.20)

[a] Experimental value: 595 nm (2.08 eV), see ref. 6 in the main text.
[b] Experimental value: 524 nm (2.37 eV), see ref. 9 in the main text.

Table S6. Cartesian coordinates (*x*, *y*, *z*, in Å) of the optimized structures for the studied complexes. The level of theory employed in each optimization is also specified. These data are also provided in the coordinates-complex1.xyz and coordinate-complex2.xyz files, which contains the coordinates for all structures in the same order as here reported for complex **1** and **2**, respectively.

Complex 1 in CH_2Cl_2

$(S_0)_{min}$ DFT B3LYP/(6-31G**+LANL2DZ) PCM

C	1.6052340	3.7630060	-1.3328240
C	1.3378870	2.5657490	-0.6544700
N	0.3569360	1.7303220	-1.1145160
C	-0.3427280	2.0609040	-2.2186620
C	2.0284700	2.0870740	0.5443810
C	1.5939120	0.8264570	1.0369860
C	2.2343000	0.3290600	2.1835380
C	3.2497070	1.0477460	2.8190620
C	3.6609880	2.2895620	2.3227690
C	3.0501130	2.8077530	1.1859210
Ir	0.0697520	-0.0063670	-0.0011670
N	0.0081050	-1.7651380	1.1126100
C	0.8088300	-2.7762320	0.6561330
C	0.8332930	-4.0028140	1.3341990
C	-0.7550350	-3.1456020	2.9187310
C	-0.7480780	-1.9520700	2.2131530
C	1.5857010	-2.4415320	-0.5386790
C	1.4067240	-1.1209030	-1.0329490
C	2.1362550	-0.7583810	-2.1767050
C	2.9966310	-1.6605940	-2.8068800
C	3.1574160	-2.9579740	-2.3082650
C	2.4518340	-3.3470280	-1.1746890
H	4.4512590	2.8452820	2.8176640
H	3.3723510	3.7716950	0.8035800
H	1.9405110	-0.6326890	2.5929390
H	3.7258460	0.6365490	3.7054120
H	2.0366270	0.2418190	-2.5872380
H	2.5788160	-4.3549170	-0.7908670
H	3.5477130	-1.3502610	-3.6908590

H	3.8273990	-3.6567870	-2.7992360
H	-1.0998830	1.3544260	-2.5328190
C	0.8816930	4.1002570	-2.4682620
H	2.3832020	4.4218530	-0.9677450
H	1.0907160	5.0261890	-2.9936860
C	-0.1134410	3.2323810	-2.9239290
H	-0.7017820	3.4521490	-3.8068040
C	0.0526810	-4.1916570	2.4662150
H	-1.3523210	-1.1098230	2.5249160
H	-1.3782580	-3.2457890	3.7993140
H	0.0739370	-5.1406300	2.9917280
H	1.4682560	-4.8016020	0.9715880
C	-2.9448910	-2.0066270	-2.6522220
C	-2.8411540	0.7671440	0.5568470
C	-3.9562180	1.4043360	1.1115740
C	-3.7865890	2.2941520	2.1679050
C	-2.5023660	2.5347320	2.6511670
C	-1.4339820	1.8745490	2.0544980
N	-1.5935510	1.0121450	1.0358520
H	-4.9490280	1.2159890	0.7251280
H	-4.6464600	2.7912540	2.6035360
H	-2.3222160	3.2194540	3.4715590
H	-0.4143930	2.0250750	2.3892110
C	-2.9352400	-0.1984580	-0.5649460
C	-4.1524940	-0.6071780	-1.1206220
C	-4.1581290	-1.5176390	-2.1730410
C	-1.7690640	-1.5642860	-2.0562910
N	-1.7586050	-0.6824770	-1.0417000
H	-5.0902780	-0.2278160	-0.7366890
H	-5.0978820	-1.8399150	-2.6079550
H	-2.9007770	-2.7175290	-3.4688780
H	-0.7979900	-1.9130210	-2.3874870

(^{3}MLCT)$_{min}$ DFT B3LYP/(6-31G**+LANL2DZ) PCM

C	1.0244470	4.0245330	1.1183260
C	0.4781370	2.8483110	0.5275950
N	0.8935940	1.6052130	1.0017940
C	1.8191210	1.5476760	1.9902640
C	2.3733840	2.6624770	2.5802910
C	1.9536580	3.9407390	2.1246990
C	-0.4768460	2.8485120	-0.5276970
N	-0.8928530	1.6055910	-1.0018710
C	-1.8183660	1.5484240	-1.9903750
C	-2.3721170	2.6634510	-2.5804520
C	-1.9518560	3.9415410	-2.1248650
C	-1.0226410	4.0249560	-1.1184640
Ir	-0.0000640	-0.1315960	-0.0000350
N	1.6959070	-0.1939250	-1.2041160
C	2.6823640	-1.0314700	-0.7680520
C	3.8800210	-1.1295350	-1.4832850
C	4.0628990	-0.3719510	-2.6348600
C	3.0441190	0.4813500	-3.0588870
C	1.8735210	0.5446890	-2.3139710
H	3.1458200	1.0915250	-3.9481950
H	1.0497550	1.1887040	-2.5928460
C	2.3528650	-1.7581540	0.4618880
C	1.0709960	-1.4703270	1.0287090
C	0.7123170	-2.1282230	2.2237770
C	1.5694060	-3.0477860	2.8199430
C	2.8156660	-3.3279470	2.2412900
C	3.2066250	-2.6861600	1.0658840
N	-1.6960730	-0.1930930	1.2040410
C	-1.8734330	0.5457880	2.3137570
C	-3.0440590	0.4830020	3.0586790
C	-4.0631360	-0.3700200	2.6348070
C	-3.8805230	-1.1278740	1.4833670
C	-2.6828340	-1.0303510	0.7681140
H	-3.1455470	1.0933790	3.9478720

H	-1.0494340	1.1895490	2.5925300
C	-2.3536030	-1.7573340	-0.4617210
C	-1.0716220	-1.4700570	-1.0285800
C	-0.7131780	-2.1282860	-2.2235440
C	-1.5706080	-3.0476100	-2.8195820
C	-2.8169790	-3.3272140	-2.2408970
C	-3.2077070	-2.6851040	-1.0655890
H	-2.3607430	4.8448720	-2.5660220
H	2.3629450	4.8439030	2.5658280
H	-2.1019630	0.5529360	-2.3113200
H	2.1022940	0.5520780	2.3112380
H	-4.6630880	-1.7914620	1.1375070
H	4.6623540	-1.7933340	-1.1373050
H	0.2460510	-1.9221100	-2.6860310
H	-0.2468270	-1.9216060	2.6862420
H	3.4829790	-4.0443170	2.7092750
H	-3.4845580	-4.0434030	-2.7087790
H	-4.1756270	-2.9162870	-0.6337730
H	-1.2755530	-3.5523620	-3.7343160
H	1.2741680	-3.5522890	3.7347550
H	4.1744530	-2.9177720	0.6340900
H	0.7014320	4.9960330	0.7640470
H	3.1047250	2.5493410	3.3714420
H	-0.6992240	4.9963290	-0.7641970
H	-3.1034760	2.5506180	-3.3716300
H	-4.9902360	-0.4429400	3.1930650
H	4.9899740	-0.4452960	-3.1931050

(^{3}MC)$_{min}$ DFT B3LYP/(6-31G**+LANL2DZ) PCM

C	-4.2794270	1.4337940	-0.9436370
C	-2.9896900	1.1298750	-0.4730600
N	-2.2360780	0.2149490	-1.1213610
C	-2.7124240	-0.4127790	-2.2014280
C	-2.3687830	1.7587940	0.7177740
C	-1.0125280	1.4436340	1.0501670
C	-0.4521180	2.0836950	2.1773440
C	-1.1818800	2.9726400	2.9635250
C	-2.5074890	3.2629360	2.6291260
C	-3.0894360	2.6630180	1.5138600
Ir	0.0002310	0.0528210	0.0000200
N	2.2369720	0.2117200	1.1216420
C	2.9924870	1.1243590	0.4723520
C	4.2827010	1.4264140	0.9428400
C	3.9810680	-0.1702360	2.7182140
C	2.7118780	-0.4155750	2.2025980
C	2.3730060	1.7530670	-0.7193220
C	1.0160750	1.4404310	-1.0513760
C	0.4571110	2.0804120	-2.1793180
C	1.1888300	2.9668140	-2.9665520
C	2.5150880	3.2545490	-2.6325280
C	3.0956800	2.6546800	-1.5165230
H	-3.0871440	3.9555860	3.2314900
H	-4.1190860	2.9078600	1.2767970
H	0.5789810	1.8703620	2.4403360
H	-0.7217800	3.4419420	3.8284160
H	-0.5744450	1.8690210	-2.4421040
H	4.1258930	2.8974450	-1.2797760
H	0.7297470	3.4361040	-3.8319900
H	3.0963040	3.9451390	-3.2357520
H	-2.0469960	-1.1359720	-2.6658020
C	-4.7738930	0.7827030	-2.0683740
H	-4.8910370	2.1748420	-0.4446680
H	-5.7681080	1.0154810	-2.4363300
C	-3.9812330	-0.1656170	-2.7171040
H	-4.3304240	-0.6952030	-3.5963660
C	4.7756830	0.7757530	2.0684750

H	2.0449500	-1.1368650	2.6677730
H	4.3290750	-0.6994090	3.5981940
H	5.7702530	1.0071020	2.4363740
H	4.8958040	2.1657330	0.4431340
C	2.3900640	-2.7817600	-2.5425920
C	-0.5081900	-2.9436960	0.5418540
C	-1.0196580	-4.1150140	1.1110620
C	-1.9715050	-4.0312500	2.1214970
C	-2.3969010	-2.7739150	2.5447610
C	-1.8484870	-1.6483320	1.9418300
N	-0.9249820	-1.7237400	0.9665690
H	-0.6786430	-5.0851740	0.7746250
H	-2.3722590	-4.9342670	2.5688470
H	-3.1366610	-2.6573830	3.3280100
H	-2.1444880	-0.6499910	2.2388770
C	0.5005490	-2.9453630	-0.5399260
C	1.0087770	-4.1183720	-1.1085740
C	1.9610020	-4.0377090	-2.1189060
C	1.8447600	-1.6543770	-1.9402150
N	0.9208480	-1.7267690	-0.9651020
H	0.6649820	-5.0874450	-0.7718020
H	2.3592800	-4.9420350	-2.5658220
H	3.1303110	-2.6676580	-3.3257380
H	2.1436930	-0.6570010	-2.2375740

(**^{3}LC-ppy)$_{min\text{-}td}$** TDDFT B3LYP/(6-31G**+LANL2DZ) PCM

Ir	0.0000000	0.0000000	0.0958720
N	0.5890980	1.2078890	-1.7161560
N	-0.5890980	-1.2078890	-1.7161560
C	0.3403690	0.6588360	-2.9310600
C	-0.3403690	-0.6588360	-2.9310600
C	0.7159250	1.3212720	-4.1048240
C	-0.7159250	-1.3212720	-4.1048240
C	1.3528080	2.5557870	-4.0241910
C	-1.3528080	-2.5557870	-4.0241910
C	1.6032650	3.1082110	-2.7702150
C	-1.6032650	-3.1082110	-2.7702150
C	1.2043900	2.3998010	-1.6420470
C	-1.2043900	-2.3998010	-1.6420470
H	1.3690130	2.7850840	-0.6426230
H	-1.3690130	-2.7850840	-0.6426230
N	-1.9643850	0.6636000	0.1889800
N	1.9643850	-0.6636000	0.1889800
C	2.5062060	-1.6388830	-0.5638860
C	-2.5062060	1.6388830	-0.5638860
C	2.7480230	-0.0142080	1.1373960
C	-2.7480230	0.0142080	1.1373960
C	3.8233620	-2.0496690	-0.4269110
C	-3.8233620	2.0496690	-0.4269110
C	4.0906860	-0.4150530	1.3178080
C	-4.0906860	0.4150530	1.3178080
C	4.6313040	-1.4242470	0.5465380
C	-4.6313040	1.4242470	0.5465380
H	-1.8460320	2.1070160	-1.2835780
H	1.8460320	-2.1070160	-1.2835780
H	4.6944900	0.0807830	2.0685440
H	-4.6944900	-0.0807830	2.0685440
H	-4.2043000	2.8443690	-1.0569060
H	4.2043000	-2.8443690	-1.0569060
C	0.6921830	1.2744200	1.4620160
C	-0.6921830	-1.2744200	1.4620160
C	0.0000000	2.3222460	2.0947550
C	-0.0000000	-2.3222460	2.0947550
C	2.0764160	1.0480580	1.8329770
C	-2.0764160	-1.0480580	1.8329770
C	0.6184240	3.1259130	3.0486890
C	-0.6184240	-3.1259130	3.0486890
C	2.6831560	1.8626010	2.8197180

C	-2.6831560	-1.8626010	2.8197180
C	1.9660300	2.8860110	3.4117620
C	-1.9660300	-2.8860110	3.4117620
H	-1.0378860	2.5096490	1.8396010
H	1.0378860	-2.5096490	1.8396010
H	-2.4407370	-3.5117100	4.1615630
H	2.4407370	3.5117100	4.1615630
H	3.7138960	1.6942980	3.1147050
H	0.0689190	3.9339440	3.5217490
H	-0.0689190	-3.9339440	3.5217490
H	-3.7138960	-1.6942980	3.1147050
H	-0.5203670	-0.8816280	-5.0738990
H	-1.6482640	-3.0751950	-4.9292770
H	-2.0968760	-4.0664090	-2.6587440
H	0.5203670	0.8816280	-5.0738990
H	1.6482640	3.0751950	-4.9292770
H	2.0968760	4.0664090	-2.6587440
H	-5.6620820	1.7310440	0.6868670
H	5.6620820	-1.7310440	0.6868670

(^{3}MLCT/^{3}MC)$_{ts}$ DFT B3LYP/(6-31G**+LANL2DZ) PCM

Ir	-0.0684170	-0.0911780	-0.0169680
N	-2.0124280	0.2015020	1.1069150
N	1.9134240	-0.5852120	-1.2890460
C	-4.2314300	-0.6814660	1.2640460
C	-3.0285910	-0.5219070	0.5628170
C	-2.1603800	0.7675690	2.3156400
C	-2.7307860	-1.1035860	-0.7554020
C	-1.3715160	-1.0463670	-1.2010000
C	-1.0675430	-1.6037350	-2.4611770
C	-2.0495550	-2.2017300	-3.2468820
C	-3.3738660	-2.2520510	-2.7925850
C	-3.7112610	-1.7063750	-1.5547100
C	2.6036660	-1.5738670	-0.6726000
C	3.9384070	-1.8247180	-1.0252490
C	3.8175780	-0.0401860	-2.6350000
C	2.5005060	0.1605190	-2.2331200
C	1.8711170	-2.3037540	0.3869260
C	0.6302850	-1.7648480	0.8573590
C	-0.0263360	-2.4506630	1.9026870
C	0.4926470	-3.6183940	2.4557410
C	1.6919270	-4.1450750	1.9634180
C	2.3721030	-3.4927240	0.9352680
H	-4.1432530	-2.7116820	-3.4051520
H	-4.7453320	-1.7445030	-1.2274970
H	-0.0432840	-1.5695410	-2.8180960
H	-1.7905440	-2.6324900	-4.2096680
H	-0.9656770	-2.0604340	2.2824230
H	3.2895640	-3.9317600	0.5571470
H	-0.0346470	-4.1244590	3.2591250
H	2.0958300	-5.0640520	2.3767650
H	-1.3116090	1.3338310	2.6855140
C	-4.3856590	-0.0919800	2.5144900
H	-5.0346410	-1.2713320	0.8389630
H	-5.3128460	-0.2146210	3.0643510
C	-3.3313830	0.6499250	3.0542170
H	-3.4124760	1.1314090	4.0220940
C	4.5459250	-1.0565140	-2.0141170
H	1.8832830	0.9345810	-2.6808260
H	4.2535300	0.5797030	-3.4104070
H	5.5787470	-1.2433330	-2.2897080
H	4.5029930	-2.6001910	-0.5221400
C	2.8766830	1.8362500	2.7522570
C	0.1838570	2.9836350	-0.3025750
C	-0.0984470	4.3038180	-0.6868740
C	-1.0075400	4.5463570	-1.7061800
C	-1.6350200	3.4613620	-2.3271890
C	-1.3313630	2.1808330	-1.8897720

N	-0.4430980	1.9359970	-0.9066670
H	0.3846460	5.1343600	-0.1886420
H	-1.2267650	5.5638620	-2.0110800
H	-2.3457570	3.6008300	-3.1332620
H	-1.7982440	1.3100170	-2.3335390
C	1.1421830	2.6381420	0.7549460
C	1.9349010	3.5898380	1.4141930
C	2.8078680	3.1915490	2.4158240
C	2.0767930	0.9388060	2.0612370
N	1.2321840	1.3155390	1.0789890
H	1.8731330	4.6348850	1.1396310
H	3.4251440	3.9232870	2.9254190
H	3.5397300	1.4757940	3.5298230
H	2.1013650	-0.1202500	2.2841260

(^{3}MC/S$_{0}$)$_{\text{stc-mecp}}$ DFT B3LYP/(ZORA-DEF2-TZVP+SARC-ZORA-TZVP)

C	3.933728	2.096314	1.055428
C	2.926353	1.322772	0.466596
N	2.515558	0.181464	1.042714
C	3.094385	-0.230711	2.168105
C	2.255235	1.711891	-0.797864
C	0.962559	1.197675	-1.123843
C	0.384648	1.611109	-2.344696
C	1.028998	2.477821	-3.211598
C	2.291623	2.962385	-2.880190
C	2.890990	2.582878	-1.684102
Ir	0.016643	-0.127465	0.031852
N	-2.591100	0.106663	-1.013262
C	-2.933740	1.288703	-0.477679
C	-3.843287	2.134816	-1.123606
C	-4.068942	0.501800	-2.856310
C	-3.148164	-0.271834	-2.161975
C	-2.294927	1.659451	0.809930
C	-0.999474	1.168243	1.163639
C	-0.461711	1.586284	2.402200
C	-1.145920	2.431566	3.259536
C	-2.410100	2.891703	2.900638
C	-2.969860	2.510311	1.685913
H	2.814512	3.629838	-3.553485
H	3.881364	2.957234	-1.462739
H	-0.596898	1.239427	-2.608889
H	0.556272	2.775555	-4.139283
H	0.521873	1.235935	2.687469
H	-3.961418	2.865610	1.437125
H	-0.702226	2.732142	4.200475
H	-2.964370	3.541618	3.565964
H	2.725744	-1.163337	2.582334
C	4.530387	1.664106	2.229348
H	4.233246	3.036536	0.616126
H	5.307159	2.257153	2.695078
C	4.112348	0.469586	2.800913
H	4.554177	0.092351	3.713183
C	-4.414295	1.736172	-2.322417
H	-2.841072	-1.238917	-2.546941
H	-4.493390	0.148113	-3.786330
H	-5.113193	2.384551	-2.835684
H	-4.080739	3.103944	-0.708385
C	-2.360655	-2.962036	2.530653
C	0.533746	-3.108479	-0.523913
C	1.078295	-4.268238	-1.072864
C	2.023780	-4.173428	-2.079421
C	2.408168	-2.913402	-2.515084
C	1.834452	-1.800455	-1.926301
N	0.914616	-1.885671	-0.953278
H	0.770051	-5.238997	-0.715307
H	2.452819	-5.066911	-2.513535
H	3.142507	-2.784148	-3.297968
H	2.110073	-0.804033	-2.235924

C	-0.476794	-3.118384	0.545860
C	-1.028431	-4.288418	1.064955
C	-1.978947	-4.213303	2.068406
C	-1.779393	-1.837991	1.971174
N	-0.854798	-1.903858	1.001372
H	-0.723296	-5.251755	0.685257
H	-2.414879	-5.114750	2.478512
H	-3.099850	-2.847639	3.311282
H	-2.054827	-0.847940	2.300899

(^{3}LC-bpy)$_{\text{min-td}}$ TDDFT B3LYP/(6-31G**+LANL2DZ) PCM

C	-4.0997160	0.8796050	1.2233050
C	-2.8955500	0.4104930	0.5663350
N	-1.6335680	0.7827560	1.1003660
C	-1.5924070	1.5379710	2.1919310
C	-2.7375940	2.0078550	2.8573930
C	-4.0163540	1.6588680	2.3448690
C	-2.8955410	-0.4105730	-0.5663340
N	-1.6335480	-0.7828020	-1.1003690
C	-1.5923720	-1.5380100	-2.1919380
C	-2.7375490	-2.0079250	-2.8573950
C	-4.0163170	-1.6589820	-2.3448610
C	-4.0996970	-0.8797250	-1.2232950
Ir	0.0674540	-0.0000020	-0.0000030
N	0.1786360	1.7860150	-1.0697390
C	1.0791640	2.6985550	-0.5918750
C	1.2286800	3.9303640	-1.2426130
C	0.4697520	4.2241100	-2.3673200
C	-0.4422880	3.2779570	-2.8400630
C	-0.5567010	2.0739130	-2.1629330
H	-1.0541840	3.4621670	-3.7148870
H	-1.2439810	1.3059090	-2.4913780
C	1.8183500	2.2619330	0.5945230
C	1.5076330	0.9578170	1.0629280
C	2.1907330	0.5001500	2.2005570
C	3.1387280	1.2970350	2.8470860
C	3.4306640	2.5804050	2.3735700
C	2.7698250	3.0622420	1.2483290
N	0.1786860	-1.7860160	1.0697330
C	-0.5566510	-2.0739400	2.1629210
C	-0.4422000	-3.2779800	2.8400520
C	0.4698800	-4.2240990	2.3673180
C	1.2288060	-3.9303260	1.2426170
C	1.0792490	-2.6985240	0.5918760
H	-1.0540970	-3.4622120	3.7148710
H	-1.2439640	-1.3059620	2.4913580
C	1.8184280	-2.2618780	-0.5945180
C	1.5076680	-0.9577730	-1.0629250
C	2.1907570	-0.5000830	-2.2005530
C	3.1387840	-1.2969360	-2.8470760
C	3.4307640	-2.5802940	-2.3735560
C	2.7699360	-3.0621540	-1.2483170
H	-4.9163770	-2.0097210	-2.8375840
H	-4.9164210	2.0095770	2.8375990
H	-0.6011960	-1.7881390	-2.5555420
H	-0.6012350	1.7881270	2.5555280
H	1.9423120	-4.6514790	0.8642950
H	1.9421560	4.6515440	-0.8642840
H	1.9879450	0.4921920	-2.5916170
H	1.9879560	-0.4921330	2.5916180
H	4.1666530	3.1982160	2.8782290
H	4.1667770	-3.1980800	-2.8782110
H	2.9980980	-4.0601810	-0.8867460
H	3.6539110	-0.9150980	-3.7245140
H	3.6538650	0.9152140	3.7245250
H	2.9979540	4.0602790	0.8867600
H	-5.0662950	0.6065930	0.8212470

H	-2.6277620	2.6322410	3.7352700
H	-5.0662820	-0.6067480	-0.8212260
H	-2.6277040	-2.6323050	-3.7352750
H	0.5877350	-5.1777830	2.8709230
H	0.5875770	5.1777980	-2.8709230

(^{1}LC-ppy)$_{\text{min-td}}$ TDDFT B3LYP/(6-31G**+LANL2DZ) PCM

Ir	-0.0000000	0.0000000	0.1100270
N	0.6037610	1.1963370	-1.7304560
N	-0.6037610	-1.1963370	-1.7304560
C	0.3504110	0.6529830	-2.9452660
C	-0.3504110	-0.6529830	-2.9452660
C	0.7401760	1.3083140	-4.1180300
C	-0.7401760	-1.3083140	-4.1180300
C	1.3952190	2.5330920	-4.0349340
C	-1.3952190	-2.5330920	-4.0349340
C	1.6495070	3.0811990	-2.7800180
C	-1.6495070	-3.0811990	-2.7800180
C	1.2373570	2.3783640	-1.6531140
C	-1.2373570	-2.3783640	-1.6531140
H	1.4072320	2.7608350	-0.6537930
H	-1.4072320	-2.7608350	-0.6537930
N	-1.9640970	0.6628470	0.1931890
N	1.9640970	-0.6628470	0.1931890
C	2.5171140	-1.6269340	-0.5787210
C	-2.5171140	1.6269340	-0.5787210
C	2.7411560	-0.0315800	1.1543650
C	-2.7411560	0.0315800	1.1543650
C	3.8270440	-2.0388740	-0.4337560
C	-3.8270440	2.0388740	-0.4337560
C	4.0709960	-0.4369950	1.3473330
C	-4.0709960	0.4369950	1.3473330
C	4.6239240	-1.4372340	0.5648760
C	-4.6239240	1.4372340	0.5648760
H	-1.8624660	2.0807430	-1.3126620
H	1.8624660	-2.0807430	-1.3126620
H	4.6669620	0.0472920	2.1124620
H	-4.6669620	-0.0472920	2.1124620
H	-4.2157180	2.8210580	-1.0750590
H	4.2157180	-2.8210580	-1.0750590
C	0.7010590	1.2911140	1.4462290
C	-0.7010590	-1.2911140	1.4462290
C	0.0000000	2.3556150	2.0463860
C	-0.0000000	-2.3556150	2.0463860
C	2.0618750	1.0465240	1.8461460
C	-2.0618750	-1.0465240	1.8461460
C	0.6019940	3.1553900	3.0152890
C	-0.6019940	-3.1553900	3.0152890
C	2.6420760	1.8430560	2.8470200
C	-2.6420760	-1.8430560	2.8470200
C	1.9217410	2.8899310	3.4176990
C	-1.9217410	-2.8899310	3.4176990
H	-1.0249170	2.5555600	1.7533500
H	1.0249170	-2.5555600	1.7533500
H	-2.3885530	-3.5055710	4.1805630
H	2.3885530	3.5055710	4.1805630
H	3.6564340	1.6539810	3.1837930
H	0.0518300	3.9766820	3.4636900
H	-0.0518300	-3.9766820	3.4636900
H	-3.6564340	-1.6539810	3.1837930
H	-0.5423990	-0.8700920	-5.0872860
H	-1.7020360	-3.0480590	-4.9387440
H	-2.1569230	-4.0318460	-2.6662470
H	0.5423990	0.8700920	-5.0872860
H	1.7020360	3.0480590	-4.9387440
H	2.1569230	4.0318460	-2.6662470
H	-5.6514130	1.7491730	0.7157640
H	5.6514130	-1.7491730	0.7157640

(^{1}MLCT)$_{\text{min-td}}$ TDDFT B3LYP/(6-31G**+LANL2DZ) PCM

C	-1.0354570	-4.0268900	1.1021600
C	-0.4826280	-2.8475330	0.5246960
N	-0.9004530	-1.6092170	1.0052860
C	-1.8225110	-1.5570760	1.9924500
C	-2.3820670	-2.6743620	2.5758600
C	-1.9667790	-3.9481280	2.1079220
C	0.4827500	-2.8475130	-0.5246920
N	0.9005230	-1.6091790	-1.0052820
C	1.8225800	-1.5570010	-1.9924440
C	2.3821860	-2.6742630	-2.5758520
C	1.9669510	-3.9480470	-2.1079130
C	1.0356290	-4.0268470	-1.1021530
Ir	-0.0000060	0.1366740	0.0000030
N	-1.6940880	0.1938850	-1.2057440
C	-2.6833260	1.0283290	-0.7702290
C	-3.8815340	1.1236740	-1.4852670
C	-4.0619350	0.3669630	-2.6375450
C	-3.0398950	-0.4820510	-3.0624090
C	-1.8693450	-0.5427810	-2.3171000
H	-3.1391930	-1.0909650	-3.9528330
H	-1.0439150	-1.1839010	-2.5972970
C	-2.3555430	1.7570090	0.4587040
C	-1.0670830	1.4798820	1.0182350
C	-0.7071930	2.1445970	2.2100320
C	-1.5698450	3.0543980	2.8113150
C	-2.8236140	3.3208910	2.2410400
C	-3.2153720	2.6753990	1.0682440
N	1.6940730	0.1939610	1.2057500
C	1.8693550	-0.5426830	2.3171170
C	3.0399040	-0.4819040	3.0624250
C	4.0619150	0.3671380	2.6375480
C	3.8814880	1.1238270	1.4852600
C	2.6832820	1.0284330	0.7702240
H	3.1392220	-1.0908010	3.9528570
H	1.0439470	-1.1838280	2.5973230
C	2.3554720	1.7570890	-0.4587160
C	1.0670230	1.4799120	-1.0182430
C	0.7071100	2.1446000	-2.2100470
C	1.5697290	3.0544260	-2.8113400
C	2.8234880	3.3209700	-2.2410680
C	3.2152680	2.6755040	-1.0682650
H	2.3811730	-4.8540030	-2.5381480
H	-2.3809620	-4.8541020	2.5381590
H	2.1041350	-0.5631200	-2.3201290
H	-2.1041060	-0.5632070	2.3201350
H	4.6655010	1.7853930	1.1388330
H	-4.6655700	1.7852180	-1.1388510
H	-0.2576640	1.9489970	-2.6652400
H	0.2575880	1.9490350	2.6652260
H	-3.4951070	4.0300440	2.7136010
H	3.4949550	4.0301430	-2.7136360
H	4.1884600	2.8964700	-0.6433010
H	1.2742380	3.5626110	-3.7239130
H	-1.2743720	3.5626040	3.7238830
H	-4.1885720	2.8963240	0.6432790
H	-0.7187840	-4.9968350	0.7388700
H	-3.1136130	-2.5656320	3.3667890
H	0.7189970	-4.9968040	-0.7388620
H	3.1137290	-2.5655030	-3.3667780
H	4.9890200	0.4381380	3.1959970
H	-4.9890420	0.4379240	-3.1959960

Complex 2 in CH3CN

$(S_0)_{min}$ DFT B3LYP/(6-31G**+LANL2DZ) PCM

C	3.3735800	3.1820030	-0.8240460
C	2.4188910	2.1576930	-0.9135340
C	2.1169360	1.4021480	0.2587940
C	2.7880520	1.7090420	1.4501620
C	3.7229670	2.7366480	1.4706830
C	4.0402060	3.4949980	0.3494440
C	1.6817990	1.7860780	-2.1227760
N	0.7933210	0.7604860	-1.9331550
C	0.0527520	0.3106240	-2.9652140
C	0.1464880	0.8467280	-4.2404170
C	1.0435500	1.8935930	-4.4543280
C	1.8106040	2.3629000	-3.3956040
Ir	0.6878020	-0.0035060	-0.0013780
C	2.1335470	-1.3926980	-0.2583550
C	2.4402990	-2.1458450	0.9142600
C	3.4060460	-3.1598960	0.8266510
C	4.0791390	-3.4649350	-0.3452470
C	3.7569700	-2.7091290	-1.4667950
C	2.8111700	-1.6914880	-1.4480780
C	1.6962330	-1.7827410	2.1218350
N	0.7975710	-0.7663950	1.9305580
C	0.0503240	-0.3243340	2.9611760
C	0.1467900	-0.8598690	4.2364160
C	1.0538800	-1.8977390	4.4518890
C	1.8280440	-2.3587780	3.3947090
N	-1.0457370	-1.3098550	-0.3387560
C	-2.2705320	-0.7431190	-0.1707140
C	-3.4327720	-1.5003090	-0.3080120
C	-3.3820330	-2.8649940	-0.6286620
C	-2.1060120	-3.4141070	-0.7964370
C	-0.9792200	-2.6148890	-0.6420220
C	-2.2778840	0.7029660	0.1621520
N	-1.0629840	1.2809570	0.3319320
C	-1.0103570	2.5902970	0.6363310
C	-2.1444510	3.3718500	0.7875330
C	-3.4184920	2.8072740	0.6168630
C	-3.4543000	1.4467730	0.2971450
H	-0.4749940	-0.4678620	5.0322640
H	-0.6341570	0.4838840	2.7400450
H	-0.4696360	0.4482260	-5.0374420
H	-0.6239750	-0.5044130	-2.7452920
C	-4.6814850	3.6627680	0.7793080
C	-4.6767240	-3.6748850	-0.7776660
H	-0.0192050	3.0088120	0.7628920
H	0.0163220	-3.0238450	-0.7668980
H	2.5112110	3.1704220	-3.5424260
H	2.5365580	-3.1591380	3.5427970
H	2.5983220	1.1646490	2.3677260
H	2.6181230	-1.1484660	-2.3657580
H	4.8190750	-4.2542960	-0.3775450
H	4.7716720	4.2921550	0.3831610
F	3.6849150	3.9258160	-1.9140880
F	4.3579640	3.0189030	2.6287830
F	4.3980720	-2.9837760	-2.6233530
F	3.7222940	-3.9011540	1.9170180
H	-4.3928550	-1.0253120	-0.1587180
H	-1.9637970	-4.4575270	-1.0443950
H	-4.4051130	0.9580650	0.1453110
H	-2.0188690	4.4190250	1.0358460
H	1.1469050	2.3407850	-5.4373830
H	1.1595730	-2.3442680	5.4349970
C	-5.9698010	2.8534310	0.5437890
C	-4.7141330	4.2368430	2.2167090
C	-4.6347490	4.8254910	-0.2415750

H	-5.5264960	5.4502350	-0.1292740
H	-3.7596350	5.4648140	-0.0950780
H	-4.6115170	4.4469840	-1.2683630
H	-6.8362530	3.5088770	0.6696130
H	-6.0142530	2.4406470	-0.4692300
H	-6.0733990	2.0309420	1.2589370
H	-5.6065490	4.8575060	2.3452710
H	-4.7478220	3.4343560	2.9603720
H	-3.8410250	4.8603790	2.4288940
C	-4.3976930	-5.1475200	-1.1299310
C	-5.4595940	-3.6262990	0.5568640
C	-5.5343940	-3.0514360	-1.9055720
H	-6.4628040	-3.6200170	-2.0190510
H	-5.8044770	-2.0134020	-1.6911920
H	-5.0027000	-3.0736810	-2.8620660
H	-6.3866560	-4.2002450	0.4607040
H	-4.8735300	-4.0606880	1.3728740
H	-5.7294410	-2.6045350	0.8389080
H	-5.3468210	-5.6824160	-1.2265450
H	-3.8652420	-5.2461730	-2.0812770
H	-3.8135790	-5.6497790	-0.3522500

(^{3}MLCT)$_{min}$ DFT B3LYP/(6-31G**+LANL2DZ) PCM

C	3.3150070	3.2379330	-0.8263460
C	2.3736120	2.2067580	-0.9139630
C	2.0866890	1.4523420	0.2720490
C	2.7294030	1.7834460	1.4780320
C	3.6458130	2.8223620	1.4964500
C	3.9595930	3.5656440	0.3589930
C	1.6482860	1.8151370	-2.1236730
N	0.8038690	0.7548080	-1.9354360
C	0.0528870	0.2932680	-2.9504840
C	0.1073980	0.8538930	-4.2201210
C	0.9659400	1.9299130	-4.4373050
C	1.7385700	2.4140240	-3.3866790
Ir	0.7384620	-0.0019580	-0.0014990
C	2.0931140	-1.4533450	-0.2697110
C	2.3759260	-2.2066450	0.9177760
C	3.3215070	-3.2345180	0.8352700
C	3.9732160	-3.5605460	-0.3466130
C	3.6626560	-2.8187690	-1.4857720
C	2.7426040	-1.7828070	-1.4722140
C	1.6434450	-1.8173640	2.1242400
N	0.7972840	-0.7590810	1.9326930
C	0.0404580	-0.2994910	2.9442920
C	0.0902920	-0.8605330	4.2139300
C	0.9501390	-1.9347950	4.4345180
C	1.7289330	-2.4166640	3.3873560
N	-0.9779950	-1.3060700	-0.3326310
C	-2.2257380	-0.7077900	-0.1669360
C	-3.3942190	-1.5044470	-0.3229480
C	-3.3396280	-2.8526330	-0.6148450
C	-2.0404690	-3.4251250	-0.7572730
C	-0.9260160	-2.6293170	-0.6055090
C	-2.2304960	0.6778290	0.1586190
N	-0.9875970	1.2852200	0.3218860
C	-0.9465640	2.6136190	0.5875640
C	-2.0666310	3.3958240	0.7357790
C	-3.3667630	2.8092900	0.5987360
C	-3.4108700	1.4631080	0.3136520
H	-0.5335390	-0.4568950	5.0020880
H	-0.6061650	0.5362730	2.7110670
H	-0.5119710	0.4485690	-5.0109260
H	-0.5930760	-0.5438760	-2.7205450
C	-4.6176150	3.6800740	0.7683820
C	-4.6313810	-3.6749580	-0.7694160
H	0.0420260	3.0434180	0.6987460
H	0.0658220	-3.0506490	-0.7202130

H	2.4063650	3.2485960	-3.5353170
H	2.3978640	-3.2497890	3.5389080
H	2.5298950	1.2440640	2.3956860
H	2.5458210	-1.2444650	-2.3911010
H	4.6970500	-4.3654370	-0.3750970
H	4.6805100	4.3730020	0.3914980
F	3.6352100	3.9702540	-1.9127380
F	4.2655840	3.1353070	2.6463580
F	4.2892000	-3.1298270	-2.6325680
F	3.6389080	-3.9652860	1.9236080
H	-4.3568520	-1.0247170	-0.1960540
H	-1.8983020	-4.4714420	-0.9899400
H	-4.3650330	0.9700770	0.1898600
H	-1.9394000	4.4470460	0.9631580
H	1.0351020	2.3921950	-5.4159830
H	1.0157150	-2.3974540	5.4132730
C	-5.9199400	2.8812320	0.5798140
C	-4.6212650	4.2912040	2.1908280
C	-4.5888310	4.8211600	-0.2773210
H	-5.4682750	5.4630830	-0.1577640
H	-3.7002980	5.4502380	-0.1712250
H	-4.5979560	4.4185970	-1.2956050
H	-6.7798400	3.5453480	0.7108180
H	-5.9882720	2.4453230	-0.4222540
H	-6.0114000	2.0731370	1.3129180
H	-5.5001440	4.9314540	2.3229520
H	-4.6550930	3.5061730	2.9534920
H	-3.7329390	4.9023220	2.3750190
C	-4.3409890	-5.1511380	-1.1013680
C	-5.4345700	-3.6234480	0.5521940
C	-5.4872270	-3.0796860	-1.9127120
H	-6.4105100	-3.6570850	-2.0326210
H	-5.7667200	-2.0410390	-1.7135530
H	-4.9445710	-3.1065860	-2.8634170
H	-6.3570120	-4.2066620	0.4561730
H	-4.8534390	-4.0418660	1.3806000
H	-5.7129720	-2.5998000	0.8191080
H	-5.2848970	-5.6954600	-1.2036880
H	-3.7943500	-5.2571380	-2.0439930
H	-3.7614140	-5.6402080	-0.3118090

(^{3}MC)$_{min}$ DFT B3LYP/(6-31G**+LANL2DZ) PCM

N	1.1147090	-1.2538940	0.4088540
C	2.3296890	-0.6810270	0.2250280
C	3.5064140	-1.4033350	0.4466370
C	3.4697120	-2.7368730	0.8636120
C	2.1945660	-3.2973290	1.0436580
C	1.0610220	-2.5375600	0.8093140
C	2.3205490	0.7300680	-0.2250920
C	3.4814210	1.4676310	-0.4515970
C	3.4270970	2.8040630	-0.8728680
C	2.1487430	3.3466970	-1.0485100
C	1.0241440	2.5678510	-0.8071350
N	1.0944000	1.2890180	-0.4064320
Ir	-0.6448390	0.0034160	0.0007860
C	-2.1035130	-1.3584540	0.4243970
C	-2.4236690	-2.3959410	-0.5070020
C	-3.3692170	-3.3532670	-0.1020710
C	-3.9962610	-3.3484900	1.1353300
C	-3.6629260	-2.3222550	2.0069830
C	-2.7488540	-1.3329960	1.6713450
C	-1.7575110	-2.4070580	-1.8302740
N	-0.8152390	-1.4509930	-1.9983130
C	-0.1274340	-1.3546290	-3.1407350
C	-0.3401350	-2.2100610	-4.2162050
C	-1.3152170	-3.1975500	-4.0733420
C	-2.0270080	-3.3028550	-2.8827090
C	-2.1250220	1.3408060	-0.4243360

C	-2.4586320	2.3763760	0.5047800
C	-3.4202180	3.3174600	0.0994260
C	-4.0502220	3.2991890	-1.1363440
C	-3.7031440	2.2757150	-2.0058260
C	-2.7731450	1.3016930	-1.6695040
C	-1.7893200	2.4022320	1.8264040
C	-2.0642030	3.3025890	2.8735900
C	-1.3480560	3.2120860	4.0628280
C	-0.3633500	2.2347980	4.2095950
C	-0.1454140	1.3743600	3.1391860
N	-0.8373230	1.4565390	1.9981640
H	-4.7814570	4.0536670	-1.3972160
F	-3.7841500	4.3316020	0.9233180
H	-2.5554720	0.5203310	-2.3878880
F	-4.3026330	2.2259340	-3.2140790
H	-2.5413570	-0.5503060	2.3913510
F	-3.7186240	-4.3708380	-0.9280450
F	-4.2601670	-2.2852960	3.2168280
H	-4.7148540	-4.1151170	1.3959220
H	0.6031800	0.5883580	3.1884530
H	-2.8265400	4.0569410	2.7604450
H	-1.5609200	3.9046380	4.8709700
H	0.2153080	2.1371740	5.1210210
H	0.6133130	-0.5610570	-3.1869280
H	0.2350190	-2.1008870	-5.1285390
H	-1.5240640	-3.8864380	-4.8856520
H	-2.7822220	-4.0648130	-2.7725400
H	4.4433770	0.9981390	-0.2979790
C	4.7201250	3.5931980	-1.1159210
H	2.0044010	4.3690680	-1.3713490
H	0.0279240	2.9732270	-0.9370410
H	4.4580450	-0.9178420	0.2898040
C	4.7320620	-3.5708380	1.1154440
H	2.0691820	-4.3243900	1.3650660
H	0.0701080	-2.9545340	0.9415580
C	6.0221950	-2.7695140	0.8635930
C	4.7285860	-4.0528080	2.5865500
C	4.7181750	-4.7958510	0.1687570
H	6.8881370	-3.4089390	1.0571690
H	6.0939430	-2.4241900	-0.1728370
H	6.1003010	-1.9011020	1.5256310
H	5.6093160	-5.4067460	0.3442090
H	3.8421370	-5.4299560	0.3315920
H	4.7213170	-4.4827140	-0.8801040
H	5.6209140	-4.6571650	2.7777460
H	4.7371750	-3.2046950	3.2784330
H	3.8539960	-4.6686090	2.8144890
C	4.4371940	5.0382920	-1.5655550
C	5.5401230	3.6360860	0.1964560
C	5.5432460	2.8846130	-2.2190680
H	5.3854000	5.5596170	-1.7248710
H	3.8802920	5.0713040	-2.5073790
H	3.8756020	5.5981430	-0.8109200
H	6.4655620	4.1972970	0.0332400
H	4.9786660	4.1305170	0.9953910
H	5.8150940	2.6357930	0.5431490
H	6.4697170	3.4387820	-2.3996800
H	5.8163400	1.8636510	-1.9370450
H	4.9845460	2.8398620	-3.1592150

(^{3}LC-ppy)$_{min}$ DFT B3LYP/(6-31G**+LANL2DZ) PCM

C	-3.4218140	-3.1597030	-0.7701310
C	-2.4535970	-2.1508710	-0.8844720
C	-2.1240830	-1.3936510	0.2790980
C	-2.7703660	-1.6849260	1.4871720
C	-3.7180380	-2.6998560	1.5314750
C	-4.0682880	-3.4567260	0.4189850
C	-1.7257740	-1.8053430	-2.1080460

N	-0.8158900	-0.7957640	-1.9414490
C	-0.0657890	-0.3835170	-2.9814130
C	-0.1759850	-0.9407690	-4.2464500
C	-1.1011010	-1.9671080	-4.4391510
C	-1.8754930	-2.4001030	-3.3699790
Ir	-0.6942480	0.0001860	-0.0126600
C	-2.0944620	1.3963110	-0.3003600
C	-2.4164430	2.1582490	0.9327330
C	-3.4212020	3.1886050	0.8153290
C	-4.0509630	3.4802610	-0.3577330
C	-3.7064100	2.7274390	-1.5075890
C	-2.7597950	1.7076940	-1.4831720
C	-1.7200140	1.8087770	2.0910700
N	-0.7639030	0.7539470	1.8950100
C	-0.0162170	0.3596760	2.9301770
C	-0.1199650	0.9067850	4.2063200
C	-1.0748340	1.9430950	4.4438570
C	-1.8519910	2.3772260	3.4076330
N	1.0610090	1.3056050	-0.3656540
C	2.2813610	0.7379100	-0.1780790
C	3.4462330	1.4932730	-0.3036510
C	3.4005050	2.8564200	-0.6322060
C	2.1276440	3.4052180	-0.8240730
C	0.9985910	2.6070520	-0.6814700
C	2.2835520	-0.7079760	0.1583660
N	1.0681390	-1.2861360	0.3211230
C	1.0127270	-2.5962490	0.6211570
C	2.1453500	-3.3790930	0.7758110
C	3.4204470	-2.8142780	0.6145480
C	3.4586100	-1.4529780	0.2983460
H	0.5195880	0.5322140	4.9961040
H	0.6906290	-0.4372360	2.7319800
H	0.4475080	-0.5728560	-5.0523540
H	0.6305900	0.4192540	-2.7771170
C	4.6822460	-3.6703720	0.7823510
C	4.6977320	3.6649390	-0.7657820
H	0.0209800	-3.0150220	0.7422380
H	0.0044100	3.0123540	-0.8301800
H	-2.5953570	-3.1932940	-3.5007440
H	-2.5839560	3.1575740	3.5562060
H	-2.5508070	-1.1367630	2.3954320
H	-2.5595510	1.1684120	-2.4017070
H	-4.7959120	4.2651490	-0.4088950
H	-4.8095240	-4.2438690	0.4711180
F	-3.7663340	-3.9035050	-1.8489160
F	-4.3321960	-2.9695610	2.7026140
F	-4.3360590	3.0312230	-2.6611630
F	-3.7571040	3.9099820	1.9134270
H	4.4040230	1.0189830	-0.1385940
H	1.9900980	4.4469350	-1.0816430
H	4.4105070	-0.9647780	0.1522190
H	2.0177580	-4.4269420	1.0200470
H	-1.2199190	-2.4277050	-5.4142050
H	-1.1801900	2.3749990	5.4325850
C	5.9720850	-2.8610530	0.5552270
C	4.7065550	-4.2466460	2.2190860
C	4.6407040	-4.8314390	-0.2406540
H	5.5318700	-5.4562780	-0.1245420
H	3.7649070	-5.4710740	-0.0997760
H	4.6229700	-4.4513350	-1.2669490
H	6.8375500	-3.5170520	0.6847630
H	6.0223000	-2.4466990	-0.4568840
H	6.0720980	-2.0398100	1.2722940
H	5.5979780	-4.8678890	2.3515050
H	4.7365280	-3.4453280	2.9641470
H	3.8320180	-4.8701220	2.4254690
C	4.4247390	5.1371690	-1.1244250
C	5.4630750	3.6184480	0.5789600
C	5.5693720	3.0382680	-1.8811950
H	6.4998170	3.6056190	-1.9835170

H	5.8353830	2.0003250	-1.6612860
H	5.0503110	3.0593520	-2.8446230
H	6.3914240	4.1919800	0.4936100
H	4.8666570	4.0545740	1.3864770
H	5.7291210	2.5972270	0.8664510
H	5.3755300	5.6713420	-1.2076120
H	3.9067530	5.2348150	-2.0838040
H	3.8293420	5.6408410	-0.3562620

(^{3}MLCT/^{3}MC)$_{ts}$ DFT B3LYP/(6-31G**+LANL2DZ) PCM

N	1.0569250	-1.2142680	0.3662420
C	2.2807480	-0.6460810	0.2237780
C	3.4493380	-1.3919600	0.4125170
C	3.4007050	-2.7413600	0.7689950
C	2.1188650	-3.2935270	0.9246780
C	0.9937950	-2.5143410	0.7151030
C	2.2955690	0.7891940	-0.1355010
C	3.4633710	1.5498090	-0.2055140
C	3.4281020	2.9061270	-0.5571470
C	2.1627150	3.4441020	-0.8229960
C	1.0317160	2.6436020	-0.7188390
N	1.0843540	1.3456440	-0.3862330
Ir	-0.7256430	0.0379170	-0.0254040
C	-2.0751930	-1.4326500	0.2474080
C	-2.3259350	-2.2895790	-0.8749680
C	-3.3185370	-3.2678110	-0.7319130
C	-4.0290060	-3.4680130	0.4444810
C	-3.7350770	-2.6404410	1.5235620
C	-2.7855840	-1.6333830	1.4448880
C	-1.5169370	-2.0976740	-2.0955590
N	-0.7780850	-0.9564720	-2.1087240
C	0.0448310	-0.6951010	-3.1346210
C	0.1777060	-1.5499870	-4.2228830
C	-0.5760730	-2.7241400	-4.2317260
C	-1.4276150	-3.0015050	-3.1658450
N	-0.8394980	1.0854490	1.9421740
C	-1.9434080	1.9018340	2.0177650
C	-2.4741210	2.2687650	3.2618980
C	-1.8829810	1.8041420	4.4331010
C	-0.7602910	0.9688310	4.3413110
C	-0.2776120	0.6339030	3.0867530
C	-2.4868980	2.2587360	0.7074370
C	-2.0770270	1.4565260	-0.4135560
C	-2.5822300	1.7484780	-1.6970660
C	-3.4715890	2.7960140	-1.8613350
C	-3.8916540	3.5930340	-0.7956150
C	-3.3898020	3.3014730	0.4641820
H	-4.5798650	4.4166110	-0.9408090
F	-3.7958310	4.0985390	1.4806890
H	-2.2912950	1.1579360	-2.5572770
F	-3.9667620	3.0636170	-3.0878540
H	-2.6104390	-1.0086920	2.3134350
F	-3.6432370	-4.0760260	-1.7685170
F	-4.4070180	-2.8310970	2.6761840
H	-4.7859610	-4.2397990	0.5085240
H	0.5945080	-0.0006410	2.9685910
H	-3.3465120	2.9058620	3.3022270
H	-2.2932850	2.0787390	5.3987400
H	-0.2574970	0.5968610	5.2272580
H	0.5985440	0.2366750	-3.0753980
H	0.8459850	-1.2950360	-5.0374620
H	-0.4999920	-3.4231850	-5.0580190
H	-2.0083050	-3.9116360	-3.1559480
H	4.4121850	1.0842640	0.0243110
C	4.7256800	3.7220710	-0.6331320
H	2.0332640	4.4798370	-1.1075910
H	0.0453340	3.0484550	-0.9114600
H	4.4047930	-0.9095300	0.2702610

C	4.6543290	-3.5980200	0.9848670
H	1.9795660	-4.3312220	1.2035030
H	0.0012550	-2.9310020	0.8273610
C	5.9521100	-2.8024080	0.7548840
C	4.6511740	-4.1301290	2.4386710
C	4.6234870	-4.7896070	-0.0028330
H	5.5362640	-4.7522520	2.6051770
H	4.6738070	-3.3064110	3.1591650
H	3.7692960	-4.7417580	2.6493440
H	6.8120170	-3.4578650	0.9196960
H	6.0214210	-2.4200800	-0.2686700
H	6.0437980	-1.9595330	1.4475260
H	5.5086250	-5.4157870	0.1474410
H	3.7413310	-5.4195330	0.1421520
H	4.6256060	-4.4407900	-1.0403690
C	4.4624050	5.1882020	-1.0229340
C	5.4217140	3.6972210	0.7491830
C	5.6578070	3.0888070	-1.6945280
H	5.4127400	5.7282350	-1.0629000
H	3.9943490	5.2711550	-2.0089870
H	3.8244110	5.6964040	-0.2928150
H	6.5891900	3.6606750	-1.7556120
H	5.9176120	2.0550170	-1.4488780
H	5.1890470	3.0954730	-2.6836410
H	6.3490140	4.2771790	0.7048020
H	4.7815140	4.1375810	1.5201030
H	5.6803820	2.6815040	1.0618690

(^{3}MC/S$_0$)$_{stc}$ DFT B3LYP/(ZORA-DEF2-TZVP+SARC-ZORA-TZVP)

N	-1.250076	1.237826	0.410691
C	-2.463482	0.694140	0.193006
C	-3.631135	1.438843	0.347952
C	-3.590278	2.769288	0.753077
C	-2.316362	3.294699	0.994021
C	-1.193813	2.518466	0.805947
C	-2.450228	-0.716358	-0.232477
C	-3.593845	-1.494103	-0.355156
C	-3.526184	-2.831859	-0.749305
C	-2.249360	-3.320839	-1.019813
C	-1.143152	-2.503662	-0.874663
N	-1.224055	-1.226691	-0.483171
Ir	0.508992	0.028245	-0.045486
C	1.832010	1.366496	0.666155
C	2.366992	2.418335	-0.146402
C	3.211955	3.334758	0.477295
C	3.566480	3.273217	1.816506
C	3.047472	2.236724	2.570556
C	2.193615	1.299582	2.025104
C	2.034618	2.508099	-1.589208
N	0.887712	1.916102	-1.962946
C	0.511579	1.958224	-3.238370
C	1.258435	2.578590	-4.231093
C	2.462817	3.161714	-3.864331
C	2.858189	3.129847	-2.535354
C	1.849953	-1.323382	-0.696844
C	2.356093	-2.393736	0.110684
C	3.222742	-3.298094	-0.503047
C	3.634141	-3.206179	-1.824373
C	3.157284	-2.143519	-2.568367
C	2.283794	-1.219054	-2.034651
C	1.988052	-2.531151	1.545334
C	2.884632	-3.007492	2.509894
C	2.478719	-3.085817	3.833741
C	1.192620	-2.690281	4.172171
C	0.374592	-2.206647	3.159275
N	0.757148	-2.119949	1.886751
H	4.303837	-3.942832	-2.245766
F	3.689692	-4.363859	0.177632

H	1.945964	-0.407144	-2.662182
F	3.562148	-2.015768	-3.841631
H	1.814440	0.511135	2.659580
F	3.722421	4.374917	-0.213461
F	3.387785	2.151127	3.865917
H	4.223451	4.016601	2.245803
H	-0.632979	-1.869085	3.379432
H	3.886075	-3.296487	2.235257
H	3.163424	-3.447649	4.590358
H	0.833945	-2.742541	5.191366
H	-0.427731	1.470063	-3.476313
H	0.910478	2.590809	-5.255005
H	3.094054	3.637803	-4.603838
H	3.795431	3.576278	-2.242815
H	-4.551802	-1.054088	-0.125008
C	-4.794975	-3.677459	-0.869438
H	-2.089737	-4.339639	-1.338571
H	-0.152693	-2.882256	-1.073935
H	-4.577863	0.973412	0.128888
C	-4.837422	3.635863	0.926700
H	-2.181574	4.318106	1.314177
H	-0.207270	2.926664	0.964734
C	-6.126088	2.881223	0.571200
C	-4.919430	4.095141	2.398817
C	-4.722860	4.870935	0.006841
H	-6.982061	3.543804	0.703148
H	-6.130977	2.548276	-0.469204
H	-6.284949	2.014178	1.216147
H	-5.606150	5.500705	0.124156
H	-3.850180	5.480188	0.244972
H	-4.654414	4.577261	-1.042518
H	-5.802887	4.719481	2.542121
H	-4.994963	3.241937	3.075811
H	-4.048550	4.682700	2.692257
C	-4.481040	-5.139381	-1.220120
C	-5.554889	-3.649662	0.474363
C	-5.684867	-3.081363	-1.981882
H	-5.411320	-5.704626	-1.283601
H	-3.979193	-5.231791	-2.185343
H	-3.859573	-5.616996	-0.459677
H	-6.458939	-4.255862	0.398452
H	-4.943493	-4.056783	1.282010
H	-5.859951	-2.640800	0.756179
H	-6.599621	-3.668852	-2.077105
H	-5.972947	-2.050647	-1.767328
H	-5.173127	-3.096094	-2.946145

(^{3}MLCT/^{3}LC-ppy)$_{ts}$ DFT B3LYP/(6-31G**+LANL2DZ)

C	-3.4576290	-3.0821590	-0.9795390
C	-2.4876420	-2.0680960	-1.0277530
C	-2.1420280	-1.4031100	0.1867930
C	-2.7730120	-1.7829910	1.3762990
C	-3.7245310	-2.7976990	1.3575040
C	-4.0872720	-3.4668880	0.1926990
C	-1.7879680	-1.6215620	-2.2314340
N	-0.8774250	-0.6241990	-2.0031530
C	-0.1560830	-0.1209500	-3.0231990
C	-0.2956770	-0.5685570	-4.3274950
C	-1.2219460	-1.5813500	-4.5834010
C	-1.9665480	-2.1084580	-3.5369230
Ir	-0.7115900	0.0066950	-0.0158700
C	-2.0785430	1.4432350	-0.1596520
C	-2.3030420	2.1611590	1.1155770
C	-3.2355260	3.2521030	1.0981310
C	-3.9194600	3.6175870	-0.0245760
C	-3.6960890	2.8839600	-1.2207810
C	-2.8053660	1.8254900	-1.2913630
C	-1.5968800	1.6890940	2.2394490

N	-0.7347250	0.5970840	1.9525950
C	-0.0283390	0.0488080	2.9511510
C	-0.0923730	0.4937110	4.2656380
C	-0.9507010	1.5798000	4.5865550
C	-1.6867300	2.1618210	3.5856190
N	1.0561630	1.3137700	-0.2957840
C	2.2747630	0.7121560	-0.1638410
C	3.4512990	1.4519590	-0.3406120
C	3.4245280	2.8195730	-0.6300390
C	2.1514850	3.4042000	-0.7458340
C	1.0150950	2.6247220	-0.5825790
C	2.2601820	-0.7296980	0.1345630
N	1.0342030	-1.3221060	0.1941120
C	0.9711800	-2.6518010	0.4017320
C	2.0926940	-3.4417050	0.5782100
C	3.3778240	-2.8613840	0.5548860
C	3.4274010	-1.4869110	0.3273150
H	0.5089690	0.0038960	5.0221500
H	0.6079960	-0.7847940	2.6790270
H	0.3038930	-0.1294310	-5.1161840
H	0.5407620	0.6664040	-2.7652650
C	4.6270220	-3.7254110	0.7695170
C	4.7317510	3.6050770	-0.8100250
H	-0.0255860	-3.0766470	0.4354670
H	0.0239410	3.0532070	-0.6840170
H	-2.6885320	-2.8908540	-3.7144620
H	-2.3545850	2.9834100	3.7990000
H	-2.5481690	-1.3031180	2.3209020
H	-2.6897030	1.3109100	-2.2376210
H	-4.6188540	4.4447630	-0.0052820
H	-4.8309020	-4.2535880	0.1949040
F	-3.8130370	-3.7375290	-2.1061690
F	-4.3199930	-3.1513860	2.5058900
F	-4.3795160	3.2619030	-2.3091280
F	-3.4504590	3.9558420	2.2303740
H	4.4057990	0.9495510	-0.2546310
H	2.0289700	4.4576530	-0.9598300
H	4.3846290	-0.9867700	0.2992590
H	1.9579940	-4.5043370	0.7414730
H	-1.3647110	-1.9570570	-5.5914490
H	-1.0272870	1.9386090	5.6065590
C	5.9258750	-2.9010280	0.7077550
C	4.5382540	-4.4017490	2.1595410
C	4.6802270	-4.8125730	-0.3317750
H	5.5623130	-5.4437560	-0.1870400
H	3.8016050	-5.4635700	-0.3104340
H	4.7449020	-4.3643570	-1.3282850
H	6.7836040	-3.5601030	0.8668400
H	6.0614450	-2.4212360	-0.2673370
H	5.9601650	-2.1298920	1.4846430
H	5.4187340	-5.0306180	2.3228230
H	4.5019420	-3.6570390	2.9610150
H	3.6551030	-5.0404670	2.2509200
C	4.4733400	5.0935090	-1.1094030
C	5.5674180	3.5028580	0.4891140
C	5.5302620	2.9973070	-1.9888170
H	6.4653860	3.5486390	-2.1269860
H	5.7887250	1.9484290	-1.8147820
H	4.9633020	3.0555110	-2.9233110
H	6.5020130	4.0602710	0.3738540
H	5.0261820	3.9233450	1.3423720
H	5.8287260	2.4683510	0.7312680
H	5.4284600	5.6126840	-1.2266090
H	3.9101400	5.2337650	-2.0376970
H	3.9312600	5.5871090	-0.2963940

(^{3}LC-bpy)$_{min-td}$ TDDFT B3LYP/(6-31G**+LANL2DZ) PCM

C	3.4225550	3.1673440	-0.7328830
C	2.4506820	2.1626300	-0.8505770
C	2.1337450	1.3850480	0.3041930
C	2.8007000	1.6500680	1.5074190
C	3.7491270	2.6634580	1.5573350
C	4.0842870	3.4408240	0.4533010
C	1.7160240	1.8283770	-2.0709010
N	0.8147420	0.8084730	-1.9108590
C	0.0609040	0.3992910	-2.9500420
C	0.1608710	0.9711570	-4.2087850
C	1.0746820	2.0093100	-4.3956340
C	1.8515730	2.4383640	-3.3273720
Ir	0.6937450	-0.0007190	-0.0030430
C	2.1266020	-1.4030700	-0.2832250
C	2.4013680	-2.1929530	0.8738670
C	3.3618590	-3.2103300	0.7745290
C	4.0508680	-3.4858070	-0.3958350
C	3.7566410	-2.6963340	-1.5024880
C	2.8219940	-1.6692840	-1.4698550
C	1.6424810	-1.8550710	2.0792690
N	0.7630660	-0.8188750	1.9062560
C	-0.0053610	-0.4021520	2.9313790
C	0.0556810	-0.9841300	4.1882040
C	0.9447430	-2.0408470	4.3874670
C	1.7379210	-2.4764650	3.3336120
N	-1.0105400	-1.2982580	-0.3656190
C	-2.2664880	-0.6816680	-0.1918900
C	-3.4604830	-1.4669010	-0.3765040
C	-3.4136440	-2.8193130	-0.6487600
C	-2.1197390	-3.4027170	-0.7521960
C	-0.9777460	-2.6053280	-0.6063700
C	-2.2658560	0.6725600	0.1792030
N	-0.9993520	1.2957070	0.3283480
C	-0.9679250	2.6122420	0.5254870
C	-2.1097660	3.4046220	0.6651440
C	-3.4163100	2.8078060	0.6095320
C	-3.4639880	1.4570480	0.3759320
H	-0.5783320	-0.6117270	4.9836890
H	-0.6721240	0.4233460	2.7197580
H	-0.4629460	0.6059050	-5.0155650
H	-0.6269240	-0.4110690	-2.7475420
C	-4.6580180	3.6819210	0.8120820
C	-4.7077510	-3.6309250	-0.8303750
H	0.0175010	3.0585390	0.6034380
H	0.0080690	-3.0454160	-0.7108880
H	2.5632980	3.2396040	-3.4535410
H	2.4318370	-3.2914720	3.4701170
H	2.5952510	1.0866510	2.4097740
H	2.6487980	-1.0970210	-2.3733940
H	4.7849210	-4.2802590	-0.4382660
H	4.8283770	4.2249610	0.5102480
F	3.7544250	3.9278160	-1.8026050
F	4.3804630	2.9114080	2.7227700
F	4.4148810	-2.9451200	-2.6528060
F	3.6554010	-3.9826610	1.8470390
H	-4.4165150	-0.9677480	-0.2930090
H	-1.9829510	-4.4532110	-0.9674340
H	-4.4153850	0.9481220	0.3262930
H	-1.9798750	4.4636110	0.8481480
H	1.1825050	2.4813830	-5.3663670
H	1.0218010	-2.5220080	5.3566750
C	-5.9622420	2.8701330	0.7193500
C	-4.5879120	4.3435230	2.2101090
C	-4.6830810	4.7815420	-0.2773530
H	-5.5581570	5.4241440	-0.1357780
H	-3.7944320	5.4184110	-0.2399550
H	-4.7425100	4.3409860	-1.2777560
H	-6.8173840	3.5358940	0.8684250
H	-6.0785370	2.3972420	-0.2612010

H	-6.0124330	2.0911080	1.4869900
H	-5.4632930	4.9833740	2.3620800
H	-4.5775310	3.5872780	3.0015930
H	-3.6969420	4.9674490	2.3260980
C	-4.4211320	-5.1129530	-1.1376100
C	-5.5427210	-3.5530800	0.4700410
C	-5.5224290	-3.0366860	-2.0038590
H	-6.4462380	-3.6078890	-2.1428140
H	-5.7995920	-1.9941820	-1.8227230
H	-4.9535540	-3.0783810	-2.9382850
H	-6.4675320	-4.1279200	0.3546460
H	-4.9884700	-3.9678190	1.3181800
H	-5.8181560	-2.5236430	0.7168090
H	-5.3677020	-5.6476490	-1.2597120
H	-3.8512370	-5.2356410	-2.0642670
H	-3.8702010	-5.5994140	-0.3262780

(^{1}LC-ppy)$_{\textbf{min-td}}$ TDDFT B3LYP/(6-31G**+LANL2DZ) PCM

C	-3.5185200	-3.0484090	-0.8138010
C	-2.4983020	-2.0927990	-0.9239960
C	-2.0837210	-1.4232020	0.2829070
C	-2.6690680	-1.7716540	1.5153500
C	-3.6508300	-2.7465330	1.5481560
C	-4.0997110	-3.3967160	0.3955940
C	-1.8029920	-1.7162760	-2.1431130
N	-0.8371210	-0.7481580	-1.9446710
C	-0.0536420	-0.3634660	-2.9745540
C	-0.1925630	-0.8820440	-4.2498210
C	-1.1917200	-1.8411890	-4.4813490
C	-1.9917890	-2.2552210	-3.4264710
Ir	-0.7273630	-0.0056970	-0.0037540
C	-2.0457950	1.4609800	-0.2766110
C	-2.4280470	2.1292360	0.9466430
C	-3.4328420	3.1073660	0.8445360
C	-4.0120650	3.4808230	-0.3569390
C	-3.5890930	2.8320880	-1.5213800
C	-2.6321360	1.8308970	-1.4999900
C	-1.7388250	1.7234780	2.1481330
N	-0.7851390	0.7249530	1.9265080
C	-0.0044350	0.3124380	2.9553010
C	-0.1257880	0.8129510	4.2337540
C	-1.1118540	1.7962830	4.4909810
C	-1.9048380	2.2399630	3.4473370
N	1.0912370	1.3080670	-0.3058760
C	2.3111120	0.7333360	-0.1608060
C	3.4773020	1.4869860	-0.2843210
C	3.4300250	2.8616920	-0.5591430
C	2.1552370	3.4214130	-0.6998560
C	1.0263360	2.6213850	-0.5675000
C	2.3099380	-0.7198560	0.1318590
N	1.0932400	-1.2971140	0.2641130
C	1.0295960	-2.6170380	0.5180190
C	2.1579130	-3.4084210	0.6558150
C	3.4367140	-2.8422220	0.5307130
C	3.4820460	-1.4711960	0.2623960
H	0.5231230	0.4395600	5.0170940
H	0.7192660	-0.4585130	2.7210030
H	0.4578840	-0.5335210	-5.0430290
H	0.6858280	0.3953230	-2.7520060
C	4.6938810	-3.7067020	0.6867970
C	4.7268210	3.6707670	-0.6905120
H	0.0366010	-3.0375490	0.6179940
H	0.0319120	3.0368500	-0.6789550
H	-2.7591140	-2.9995370	-3.5791360
H	-2.6573740	2.9971150	3.6151720
H	-2.3710370	-1.2887580	2.4373650
H	-2.3554520	1.3473760	-2.4288740
H	-4.7726410	4.2515490	-0.3848180

H	-4.8774990	-4.1490170	0.4378350
F	-3.9788090	-3.6839230	-1.9150900
F	-4.2085350	-3.0889170	2.7262990
F	-4.1429510	3.2035460	-2.6970280
F	-3.8776960	3.7399460	1.9582010
H	4.4360580	1.0019460	-0.1616930
H	2.0157680	4.4732450	-0.9109000
H	4.4364960	-0.9794770	0.1487260
H	2.0238110	-4.4637360	0.8608140
H	-1.3380470	-2.2592070	-5.4712580
H	-1.2443520	2.1996890	5.4886580
C	5.9883240	-2.8941710	0.5011590
C	4.6983910	-4.3276230	2.1051320
C	4.6619970	-4.8350640	-0.3725760
H	5.5512050	-5.4642410	-0.2657250
H	3.7841800	-5.4778010	-0.2615410
H	4.6564300	-4.4231990	-1.3866420
H	6.8503390	-3.5567010	0.6196630
H	6.0516270	-2.4474240	-0.4963150
H	6.0820520	-2.0969790	1.2456200
H	5.5867020	-4.9550120	2.2289350
H	4.7208380	-3.5500830	2.8752020
H	3.8199320	-4.9549800	2.2807870
C	4.4517660	5.1555540	-0.9909760
C	5.5184560	3.5735580	0.6362500
C	5.5735270	3.0813240	-1.8447350
H	6.5034620	3.6496920	-1.9456690
H	5.8407700	2.0357560	-1.6666250
H	5.0354450	3.1374400	-2.7962190
H	6.4468850	4.1472210	0.5530380
H	4.9396800	3.9818110	1.4706890
H	5.7864360	2.5417950	0.8810830
H	5.4026440	5.6890550	-1.0772080
H	3.9123470	5.2885830	-1.9341520
H	3.8763400	5.6339960	-0.1921320

(^{1}MLCT)$_{min-td}$ TDDFT B3LYP/(6-31G**+LANL2DZ) PCM

C	3.2884560	3.2482380	-0.8792720
C	2.3615240	2.2028250	-0.9517980
C	2.0913420	1.4545440	0.2432440
C	2.7402480	1.8036720	1.4414780
C	3.6423790	2.8539020	1.4442730
C	3.9366440	3.5936030	0.2983200
C	1.6357860	1.7890550	-2.1533950
N	0.8022240	0.7233860	-1.9480400
C	0.0574780	0.2387400	-2.9563680
C	0.1055110	0.7813900	-4.2343690
C	0.9515880	1.8635890	-4.4680180
C	1.7192880	2.3709960	-3.4249460
Ir	0.7471050	-0.0025300	-0.0016740
C	2.1043970	-1.4495660	-0.2441910
C	2.3789790	-2.1943480	0.9517540
C	3.3150110	-3.2319050	0.8815970
C	3.9679100	-3.5728800	-0.2946340
C	3.6688970	-2.8368700	-1.4415880
C	2.7576080	-1.7943620	-1.4410560
C	1.6479000	-1.7858720	2.1520540
N	0.8055630	-0.7274720	1.9448990
C	0.0553150	-0.2481710	2.9517790
C	0.1061190	-0.7894490	4.2302060
C	0.9609430	-1.8643930	4.4657520
C	1.7343870	-2.3661280	3.4241960
N	-0.9738740	-1.3162440	-0.3220340
C	-2.2183960	-0.7134020	-0.1614270
C	-3.3879310	-1.5076710	-0.3166240
C	-3.3357640	-2.8549150	-0.6167350
C	-2.0395770	-3.4289410	-0.7680980
C	-0.9245190	-2.6340810	-0.6101600
C	-2.2247420	0.6774220	0.1526300

N	-0.9869270	1.2908680	0.3160670
C	-0.9517800	2.6119950	0.6053870
C	-2.0742550	3.3906670	0.7602390
C	-3.3697140	2.8015740	0.6053730
C	-3.4081700	1.4578140	0.3050200
H	-0.5134230	-0.3701450	5.0135260
H	-0.5880030	0.5869730	2.7084630
H	-0.5094550	0.3574590	-5.0188010
H	-0.5793370	-0.6019430	-2.7151300
C	-4.6250120	3.6667610	0.7729860
C	-4.6298000	-3.6740430	-0.7693980
H	0.0350400	3.0440910	0.7234950
H	0.0667300	-3.0565790	-0.7262060
H	2.3785900	3.2099310	-3.5860700
H	2.4004420	-3.1993940	3.5869330
H	2.5556130	1.2681820	2.3643260
H	2.5696830	-1.2613280	-2.3646960
H	4.6842960	-4.3844460	-0.3142930
H	4.6459950	4.4112770	0.3198360
F	3.5890490	3.9776320	-1.9729990
F	4.2678110	3.1848730	2.5856880
F	4.2987740	-3.1633420	-2.5819380
F	3.6202210	-3.9576290	1.9765830
H	-4.3500090	-1.0287030	-0.1847990
H	-1.8993820	-4.4742400	-1.0047830
H	-4.3604430	0.9644920	0.1703640
H	-1.9516330	4.4398190	0.9976260
H	1.0153120	2.3121270	-5.4534000
H	1.0270290	-2.3116870	5.4515530
C	-5.9234790	2.8660430	0.5664130
C	-4.6420950	4.2644470	2.2008290
C	-4.5909930	4.8171980	-0.2620900
H	-5.4737320	5.4549780	-0.1449590
H	-3.7055880	5.4482240	-0.1423790
H	-4.5893570	4.4236580	-1.2839630
H	-6.7862620	3.5264450	0.6970120
H	-5.9827760	2.4396230	-0.4403150
H	-6.0183990	2.0506370	1.2909550
H	-5.5242930	4.9002000	2.3321730
H	-4.6789430	3.4721600	2.9558420
H	-3.7574320	4.8768920	2.3977540
C	-4.3437890	-5.1486850	-1.1117800
C	-5.4254140	-3.6291280	0.5569090
C	-5.4907190	-3.0706400	-1.9044560
H	-6.4153270	-3.6460380	-2.0231800
H	-5.7678200	-2.0328480	-1.6975860
H	-4.9529680	-3.0924080	-2.8580920
H	-6.3491380	-4.2105100	0.4627530
H	-4.8401240	-4.0531390	1.3795430
H	-5.7008530	-2.6066850	0.8314580
H	-5.2891090	-5.6907920	-1.2125450
H	-3.8020810	-5.2497690	-2.0577860
H	-3.7608970	-5.6434450	-0.3282290
